# Supplementary material for: Proteomics and personalized PDX models identify treatment for a progressive malignancy within an actionable timeframe
Source: EMBO Mol Med. 2025 Apr 1;17(4):625–44. doi: 10.1038/s44321-025-00212-8 (PMC11982353; doi:10.1038/s44321-025-00212-8)
Supplement: Supplementary file 1 — Appendix [file 44321_2025_212_MOESM1_ESM.pdf]

Proteomics and personalized PDX models identify treatment for a progressive malignancy within an actionable timeframe

Georgina D. Barnabas[1,3*], Tariq A. Bhat[2,3*], Verena Goebeler[2,3], Pascal Leclair[2,3], Nadine Azzam[4], Nicole Melong[4], Colleen Anderson[5,6], Alexis Gom[5,7], Seohee An[2,3], Enes K. Ergin[1,3], Yaoqing Shen[8], Agustina Conrrero[1], Andrew J. Mungall[8], Karen L. Mungall[8], Christopher A. Maxwell[2,3], Gregor S.D. Reid[2,3], Martin Hirst[8,9], Steven Jones[8,9], Jennifer A. Chan[5,6,12], Donna L. Senger[10,11], Jason N. Berman[4,13], Seth J. Parker[14,15], Jonathan W. Bush[1], Caron Strahlendorf[2], Rebecca J. Deyell[2,3#], C. James Lim[2,3#], Philipp F. Lange[1,3,16#]

Appendix Table of Contents

Appendix Supplementary Information....2-5

R1 Genome Profiling 2

R3 Genome Profiling 2

R3 Proteome Profiling and Proteogenomics.... 2

One Carbon Metabolism 3

PDX Models: Mapping Tumor Histology through SETTLE PDX Models and SHMT2 Expression Analysis 3

Drug Combinations: Sertraline and Artemether Combination as a Potential Therapeutic Approach for SETTLE Tumors 4

References:.... 5

Appendix Table S1: Genome and Transcriptome analysis of SETTLE relapses 5

Appendix Figure S1: Proteogenomics of R1 and R3 8

Appendix Figure S2: Pathway analysis of the primary tumor and lung metastases.... 11

Appendix Figure S3: Characterization of SETTLE patient-derived mouse xenograft (NSG-PDX) model..... 11

Appendix Figure S4: Characterization of SETTLE patient derived chorioallantoic membrane xenograft (CAM-PDX) model. 12

Appendix Figure S5: Characterization of zebrafish-PDX model of SETTLE..... 12

Appendix Figure S6: *In vitro* viability assays of SETTLE and breast cancer cell lines..... 13

R1 Genome Profiling

On a routine follow-up visit, a chest x-ray showed multiple bilateral pulmonary nodules. A re-staging chest computed tomography (CT) showed multiple, bilateral pulmonary nodules up to 20 mm with no change in the right hemithyroidectomy surgical bed. A positron emission tomography/CT showed mildly avid pulmonary nodules, but no local recurrence or other distant metastatic disease. An infrared-guided video-assisted thoracoscopic surgery wedge resection of a pulmonary nodule confirmed SETTLE recurrence.

Whole genome analysis of the thoracoscopic biopsy of relapse (R1) showed a heterozygous variant of uncertain significance (VUS) p.L780F in FLT3 and a subclonal VUS in MUC4. The genome appeared stable with no indication of microsatellite instability or homologous recombination deficiency and no detectable somatic copy number variation or loss of heterozygosity that affected coding regions. Multiple cancer-related genes showed high RNA expression including ROS1, FGFR1, EGFR, and PDGFA in kinase signaling, SMO, GLI2, and GLI3 in the hedgehog pathway, as well as IRS4, HES4, and MYCN. Among those involved in cell cycle regulation, MDM4 showed high RNA expression, while CDKN1A had low RNA expression (Table S1A). Based on the genomic findings, the patient was treated with Sorafenib for 20 months. The tumor remained stable until further progression was detected.

R3 Genome Profiling

Genes with differences in RNA expression level between R1 and R3 included SPINK1, MMP8, HOXB13, IGF2, HAVCR2, CA9, and MET (Table S1A, B). Several receptor tyrosine kinases showed reduced RNA expression from R1 to R3 (Fig S1A), and RNA and protein levels of FLT3 were low or below the detection limit at R1 and R3 (Table S1A, B), potentially explaining the acquired resistance to Sorafenib. As the biopsy material was too limited, we could not perform phosphopeptide enrichment to assess kinase and pathway activation status. We envision including this in future studies to expand the ability to characterize this important target group.

R3 Proteome Profiling and Proteogenomics

Our global proteome analysis across 4 disease time points, 3 metastatic sites, and two adjacent normal tissues quantified 6921. It is now well established that proteomics on FFPE tissues faithfully recapitulates the proteome with similar coverage and good correlation to fresh frozen tissue sections (Piehowski *et al*, 2018), and we previously confirmed that our ASAP FFPE proteome processing allows for unbiased proteome profiling (Barnabas *et al*, 2023). In contrast to fresh sectioning, FFPE biopsy processing is a routine element of the clinical workflow and robust to delays, interruptions, and transfer between institutions, making it the preferred sample source. Evaluation of subcellular location annotations for quantified proteins showed the typical moderate

derichment of nucleoplasm, vesicular, and plasma membrane locations and enrichment of proteins annotated to be localized to the cytoplasm, mitochondria, and endoplasmic reticulum with overall balanced coverage across all subcellular locations (Fig. S1B). The median coefficient of variation (CV) between sequential sections was between 8-32%, with a median of 16% (Fig. S1C). To estimate the depth of the expressed genes quantified at the protein level we intersected RNAseq and proteome data. We found that RNAseq quantified 56.54% (R1) and 62.42% (R3) of genes above a threshold of 0.01. Of those expressed genes, 28.71% (R1) and 28.88% (R3) were quantified at the protein level (Fig. S1D,E). Focussing on pediatric cancer-associated proteins, we found that 75.43% (R1) and 83.43% (R3) of the 350 CAP genes were quantified by RNAseq above

a threshold of 0.01. Of those expressed CAP genes 38.86% (R1) and 39.14% (R3) were quantified at protein level (Fig. S1F,G).

Differential Proteome and Pathway Analysis

Differential analysis between tumors and adjacent normal regions showed 795 upregulated proteins and 728 downregulated proteins in primary tumor and lung metastases (Fig. S2A). Pathway analysis revealed the upregulation of proteins involved in transcription regulation, histone binding, protein-DNA complex assembly, amino acid biosynthesis, and protein translation machinery in the primary tumor and lung metastases. Proteins involved in extracellular matrix (ECM) assembly, cell-substrate adhesion, cholesterol transport, antioxidant activity, and complement activation pathways were downregulated in tumors (Fig. S2B). The protein abundance of the primary tumor was very similar to that of recurrent lung metastases, revealing similar dysregulation of cellular pathways compared to the adjacent normal tissues (Fig. S2C). Primary tumor and lung metastases showed indications of proliferative signatures, including upregulation of cell cycle pathways, DNA replication, transcription, translation, and ribosome biogenesis. Conversely, ECM-receptor interaction, cell-cell adhesion, immune processes such as MHC protein complex and humoral immune response, complement activation, and antigen processing and presentation were downregulated in the primary tumor and all lung metastases. Proteins involved in Glycan and Glycosphingolipid biosynthesis were uniquely upregulated in the metastases at relapse but not the primary tumor (Fig. S2C).

One Carbon Metabolism

A close inspection of the 1C metabolism pathway revealed a subtle increase, albeit mostly statistically non-significant, in the abundance of many proteins, including SHMT1, dihydrofolate reductase (DHFR), methylenetetrahydrofolate dehydrogenase 1 (MTHFD1) and MTHFD2 (Fig S2D-G). SHMTs convert serine to glycine, thereby providing carbons for 1C metabolism to support *de novo* nucleotide and amino acid biosynthesis. MTHFD1 and MTHFD2 are integral components of the 1C metabolism pathway and exhibited increased abundance in one of the lung relapses compared to adjacent normal lung tissue. DHFR, a key enzyme upstream in the 1C metabolism pathway, demonstrated elevated levels in the recurrent lung relapses, while no notable changes were observed in the primary tumor. DHFR, a well-established target to inhibit tumor growth, catalyzes the reduction of dihydrofolate to tetrahydrofolate and antifolate medications are recognized for their ability to inhibit DHFR.

PDX Models: Mapping Tumor Histology through SETTLE PDX Models and SHMT2 Expression Analysis

To facilitate targeted drug analysis, viable cryopreserved specimens of the resected R2 were successfully grafted into a hind flank of NOD.Cg-Prkdcscid Il2rgtm1Wjl/SzJ mice (NSG-PDX) by subcutaneous injection and as onplants onto CAM (CAM-PDX). Histological investigation of NSG-PDX (Fig. S3) and CAM-PDX (Fig. S4) revealed morphological characteristics consistent with those seen in the patient-derived sample.

The NSG-PDXs of primary SETTLE required 6-8 months to establish, with the resulting tumors achieving significant expansion and cellular content of >80% human (Fig. S3). Histological examination of the NSG-PDX showed a dense collection of cells with unique round and spindle shapes, with a varied chromatin pattern characterized by irregular nuclear membranes and nucleoli. In addition, tightly packed cellular clusters resemble the patient surgical specimens, with cells

exhibiting spindle-like features and forming structures resembling follicles, but no heterologous differentiation, like intestinal epithelium, was observed (Fig. S3).

In parallel, viable cryopreserved specimens of the resected R2 were also engrafted as 'onplants' onto gently lacerated ED11 chicken chorioallantoic membrane (CAM), and growth monitored by imaging over 5-7 days (details in Methods and Materials). Histological examination of the CAM-PDX microtumors of SETTLE showed a distinct region of human cellular mass encapsulated by CAM-derived epithelia within the Matrigel scaffold (Fig. S4). Atypical cells, either in clusters or as individual cells, are evident within the tumor mass. Tumor cells within the CAM-PDX displayed a predominance of discohesive growth patterns with mild to moderate pleomorphism and hyperchromasia and often maintained a spindle or oval morphology. The nuclei often showed prominent nucleoli and irregular nuclear membranes, with occasional mitotic figures identified (Fig. S4). We noted that solid fascicular growth pattern was not observed in CAM-PDX of SETTLE tumor as compared to NSG-PDXs. We are unsure of the etiology resulting in this morphologic difference, and suggest the difference in time of engraftment for a slow growing tumor indolent SETTLE tumor between the two models (6-8 months in NSG-PDX, 5-7 days in CAM-PDX) is a possible contributor.

SETTLE cells isolated from NSG-PDX tumors were also engrafted in larval zebrafish to establish zebrafish-PDX for additional pre-clinical assessment. The presence of SETTLE cells within the zebrafish-PDX was confirmed through H&E staining which highlighted morphological traits closely resembling the histological patterns of SETTLE tumors derived from mice, including high nuclear to cytoplasmic ratio, hyperchromasia, and nuclear membrane irregularities (Fig. S5).

Drug Combinations: Sertraline and Artemether Combination as a Potential Therapeutic Approach for SETTLE Tumors

There are additional druggable opportunities in the 1C metabolic pathway that may be exploited to elicit a more effective response. Cellular metabolic reprogramming is a hallmark of cancer progression and 1C metabolism involves folate and methionine progressions to generate 1C units for the biosynthesis of imperative anabolic precursors. 1C metabolism facilitates the biosynthesis of purine and thymidine production for the high needs of dividing cancer cells. It was shown that the antiproliferative effects of sertraline in combination with the mitochondrial inhibitor artemether led to cell-cycle arrest in the G1-S phase and antitumor activity against serine-dependent cell models of breast cancers (Geeraerts *et al*, 2021). We found that combinations of sertraline and artemether exhibited drug synergy profiles in NSG-PDX cells of SETTLE comparable with those in the serine-addicted MDA-MB-468 cells, but not with the non-addicted MDA-MB-231 cells (Fig. S6).

References:

Barnabas GD, Goebeler V, Tsui J, Bush JW, Lange PF (2023) ASAP horizontal line Automated Sonication-Free Acid-Assisted Proteomes horizontal line from Cells and FFPE Tissues. *Anal Chem* 95: 3291-3299

Geeraerts SL, Kampen KR, Rinaldi G, Gupta P, Planque M, Louros N, Heylen E, De Cremer K, De Brucker K, Vereecke S *et al* (2021) Repurposing the Antidepressant Sertraline as SHMT Inhibitor to Suppress Serine/Glycine Synthesis-Addicted Breast Tumor Growth. *Mol Cancer Ther* 20: 50-63

Piehowski PD, Petyuk VA, Sontag RL, Gritsenko MA, Weitz KK, Fillmore TL, Moon J, Makhlouf H, Chuaqui RF, Boja ES *et al* (2018) Residual tissue repositories as a resource for population-based cancer proteomic studies. *Clin Proteomics* 15: 26

Appendix Table S1: Genome and Transcriptome analysis of SETTLE relapses

Whole genome and transcriptome analysis of the thoracoscopic biopsy of (**A**) relapse R3 and (**B**) relapse R1. Gene expression is compared to normal thyroid and lung tissue.

A.

Genes	Proteins	TPM	kIQR.Thyroid	Percentile.Thyroid	kIQR.Lung	Percentile.Lung	Mean.ProteinAbundance	Genomic Changes
ADRA1A	NA	8.04	45.75102	100	1.132046	87.71626	NA	
AKT1	P31749	72.19	0.197719	62.48086	-0.40515	27.85467	17868.22	
AKT2	P31751	109.03	0.366921	68.14701	8.693544	100	36012.65	
AKT3	Q9Y243	48.43	2.366142	95.55896	1.762128	95.32872	7992.775	
ARID1A	O14497	47.03	0.369983	68.75957	1.842424	98.78893	753783.6	copy loss and truncating mutation p.R1335
BRAF	P15056	29.34	2.252964	98.2389	2.290591	97.92388	90989.95	
CA9	Q16790	8.62	36.11767	100	20.79534	98.96194	66067.43	
CDKN1A	NA	16.52	-1.02037	0.153139	-0.89489	0	NA	
CDKN2A	NA	8.1	12.80145	100	6.909413	100	NA	
CTNNB1	P35222	369.02	5.032673	99.84686	4.908153	100	536292.6	
DTX1	NA	9.82	-0.22282	39.05054	2.489021	97.40484	NA	
DTX2	NA	15.63	1.333151	93.87443	0.235793	61.5917	NA	
DTX3	Q8N9I9	59.06	-0.62565	17.30475	1.399407	92.47405	136613.4	
EGFR	P00533	78.07	4.998115	100	4.289055	100	99978.65	
EIF4G3	O43432	29.62	-1.08529	5.972435	0.901961	86.15917	34279.76	
FGF8	NA	27.5	399.9723	100	191.1557	100	NA	
FGFR1	P11362	574.72	23.09476	100	11.94577	100	21864.52	
FGFR2	NA	133.82	6.487744	100	12.89403	100	NA	
FGFR3	NA	7.23	1.94692	95.40582	-0.61617	14.01384	NA	
FGFR4	NA	3.55	1.709677	95.48239	-1.27903	0.17301	NA	
FLT1	P17948	14.46	-1.00787	0.765697	-0.53137	14.53287	39413.12	
FLT3	NA	0.28	0.200729	61.8683	-0.62589	7.093426	NA	heterozygous variant of uncertain significance (VUS) p.L780F
FOLR1	P15328	53.07	0.595391	75.4977	-0.55187	19.55017	296461.2	
FZD7	O75084	40.22	4.455761	99.84686	3.942828	99.65398	39393.98	
GLI2	NA	8.42	3.741359	99.69372	1.48677	93.59862	NA	
GLI3	NA	80.7	17.29494	100	26.87133	100	NA	
HAVCR2	NA	20.02	8.268143	99.69372	-0.38015	28.02768	NA	
HES4	NA	31.42	0.426848	68.91271	0.030095	52.24913	NA	
HMGA2	P52926	5.71	48.67359	100	107.9154	100	33340.77	
HOXB13	NA	7.5	262.2378	100	431.6547	100	NA	
IGF1R	NA	13.48	-1.31652	2.603369	-0.69537	14.18685	NA	
IGF2	NA	182.19	10.42342	100	13.21534	99.82699	NA	
IGF2R	P11717	40.57	2.092958	98.46861	0.342282	67.47405	44759.13	
INS	NA	0	-0.27526	21.28637	-0.25619	22.05882	NA	

INSR	P06213	32.39	0.13503	57.27412	2.343595	98.78893	58437.27	
IRS1	P35568	17.72	-1.0077	6.125574	1.233202	88.23529	12977.89	
IRS2	Q9Y4H2	25.81	1.414718	92.95559	0.073672	53.80623	12841.14	
IRS4	O14654	82.92	65.29657	100	1182.558	100	24717.36	
KAT6B	NA	73.13	15.77958	100	12.07249	100	NA	
KDR	NA	22.72	-1.10004	1.990812	-0.91382	5.795848	NA	
KMT2C	NA	48.92	4.706376	100	5.374681	100	NA	
KMT2D	NA	67.98	3.646552	100	5.561174	100	NA	
KRAS	P01116	14.59	-0.11733	44.71669	-0.70729	10.0346	281690.5	
KRT14	P02533	388.44	113.7091	100	151.1134	100	12835147	
LGR5	NA	54.71	61.01105	100	39.65992	100	NA	
MDM2	NA	64.08	10.88535	100	6.884716	100	NA	
MDM4	NA	73.89	4.664356	100	6.779127	100	NA	
MET	P08581	32.63	1.956897	95.25268	1.821525	96.88581	14729.52	
MMP8	NA	1.66	5.246002	96.32466	-0.1601	39.79239	NA	
MMP9	P14780	87.14	22.55411	99.84686	1.694421	89.27336	4625.684	
MTOR	P42345	22.5	0.673913	82.54211	1.301216	98.44291	22503.88	
MUS81	NA	31.33	-1.125	3.598775	-0.55289	16.6955	NA	copy loss
MYCN	NA	59.58	14.08338	100	110.6119	100	NA	
NRAS	P01111	36.08	3.280374	99.84686	1.33505	91.86851	60291.57	
PDGFA	NA	64.99	5.185792	99.84686	1.072582	86.15917	NA	
PDGFD	NA	35.32	-0.19482	40.73507	7.25	99.65398	NA	
PDGFRA	NA	8.63	-1.05644	4.287902	-1.14372	0.17301	NA	
PDGFRB	P09619	40.62	-1.56229	0.765697	-1.29004	0.346021	28768.33	
PIK3CA	P42336	20.22	1.746696	95.25268	0.697869	82.35294	7330.277	
PLCG1	P19174	142.16	1.938581	95.55896	2.830684	99.48097	89245.89	
RIF1	Q5UIP0	45.49	12.28387	100	12.73125	100	12975.55	
RNF43	NA	25.7	20.65608	100	7.297874	100	NA	
ROS1	NA	29.15	1930.464	100	0.523725	66.43599	NA	
RPTOR	Q8N122	16.04	2.202073	99.38744	1.577341	98.09689	16374.38	
SF3B1	O75533	200.53	0.54	75.80398	1.020932	88.75433	415662.1	subclonal mutation :p.K700E
SHMT1	P34896	16.51	-1.31005	0.765697	0.15691	56.48789	43305.92	
SMO	NA	37.68	1.647901	94.9464	4.481889	99.65398	NA	
SPINK1	NA	14.51	23.67552	99.69372	10.73982	98.2699	NA	
TP53	NA	51.81	4.700292	100	3.822514	100	NA	
VEGFA	NA	126.42	-1.57837	0.459418	-0.28222	29.58478	NA	
VEGFD	NA	2.41	-0.19157	36.44717	-0.75454	0	NA	
WNT3A	P56704	45.2	662.3343	100	5.72801	99.82699	18289.23	
YAP1	P46937	152.83	3.532537	99.69372	2.504818	99.48097	152130.3	
ZNRF3	NA	28.07	9.404676	100	7.715443	100	NA	

B.

Genes	Proteins	TPM	kIQR.Thyroid	Percentile.Thyroid	kIQR.Lung	Percentile.Lung	Mean.ProteinAbundance	Genomic Changes
ADRA1A	NA	8.54	48.65969	100	1.263893	89.44637	NA	
AKT1	P31749	37.07	-1.47148	1.071975	-2.15961	0.346021	28060.56	
AKT2	P31751	54.57	-0.85525	5.513017	2.329535	97.75087	16636.8	
AKT3	Q9Y243	9.7	-1.44587	0.765697	-1.13954	2.595156	7448.372	
ARID1A	O14497	29.11	-1.19372	5.819296	-0.22626	37.19723	486220.5	
BRAF	P15056	13.13	-0.95059	6.967841	-0.40883	26.90311	34773.56	
CA9	Q16790	1.32	4.881044	99.38744	2.614657	90.65744	17091.75	
CDKN1A	NA	2.65	-1.1773	0	-0.96201	0	NA	
CDKN2A	NA	0.85	0.284185	64.47167	-0.22536	37.54325	NA	
CTNNB1	P35222	156.5	-0.22772	34.53292	-0.18825	40.83045	301445.4	
DTX1	NA	7.45	-0.52632	19.60184	1.635889	93.94464	NA	
DTX2	NA	8.06	-0.73912	12.86371	-1.04835	2.422145	NA	
DTX3	Q8N9I9	36.64	-1.26879	0.918836	0.133277	57.78547	52535.32	
EGFR	P00533	28.64	0.339303	66.00306	0.499137	72.83737	83548.93	
EIF4G3	O43432	14.75	-2.37946	0.153139	-1.34087	1.903114	34119.8	
FGF8	NA	29.73	432.4323	100	206.7039	100	NA	
FGFR1	P11362	213.5	7.320961	100	3.556033	99.65398	16808.91	
FGFR2	NA	45.47	0.934632	87.44257	3.91514	100	NA	
FGFR3	NA	3.72	0.189284	60.18377	-0.91305	1.384083	NA	
FGFR4	NA	1.54	-0.0914	44.86983	-1.30389	0.17301	NA	
FLT1	P17948	2.71	-1.30609	0	-1.10287	0	60046.27	
FLT3	NA	0.11	-0.27586	27.25881	-0.70789	1.557093	NA	heterozygous variant of uncertain significance (VUS) p.L780F
FOLR1	P15328	22.36	-0.30047	32.00613	-0.89266	3.806228	122437	
FZD7	O75084	20.87	1.376512	93.41501	1.339835	90.48443	31912.44	
GLI2	NA	4.85	1.613826	91.57734	0.298552	63.14879	NA	
GLI3	NA	47.66	9.057592	100	14.89598	100	NA	

HAVCR2	NA	2.94	0.219133	61.40888	-1.4844	0.17301	NA	
HES4	NA	19.69	-0.18825	40.88821	-0.34949	24.04844	NA	
HMGA2	P52926	3.92	33.17576	100	73.90119	100	24295.29	
HOXB13	NA	0.04	1.398601	84.53292	2.302158	92.04152	NA	
IGF1R	NA	5.9	-1.93328	0.306279	-1.51638	0	NA	
IGF2	NA	1.12	-1.03671	0.612557	-0.61762	1.384083	NA	
IGF2R	P11717	18.7	-0.98732	8.728943	-1.38452	1.038062	39763.46	
INS	NA	0	-0.27526	21.28637	-0.25619	22.05882	NA	
INSR	P06213	11.07	-1.04483	0.153139	-0.47931	25.60554	14873.32	
IRS1	P35568	7.73	-1.52027	0.306279	-0.49345	19.55017	3931.227	
IRS2	Q9Y4H2	17.14	0.425556	71.05666	-0.30316	31.83391	19780.5	
IRS4	O14654	92.41	72.89282	100	1317.937	100	7680.022	
KAT6B	NA	26.81	4.017268	100	3.373897	100	NA	
KDR	NA	2.57	-1.4862	0	-1.57038	0	NA	
KMT2C	NA	18.54	-0.39094	30.32159	0.212404	59.51557	NA	
KMT2D	NA	27.33	-0.24713	35.83461	0.486267	74.04844	NA	
KRAS	P01116	2.85	-3.248	0	-2.31496	0	134549.2	
KRT14	P02533	52.97	14.92521	98.77489	19.97694	99.30796	3956213	
LGR5	NA	16.77	18.22801	100	11.54997	100	NA	
MDM2	NA	15.49	0.569002	76.64625	-0.41382	28.2872	NA	
MDM4	NA	28.1	0.130693	56.8147	0.767968	81.6609	NA	
MET	NA	5.08	-1.43596	0.459418	-0.37404	21.6263	NA	
MMP8	NA	0	-0.40218	6.431853	-0.41502	0.432526	NA	
MMP9	P14780	0.42	-0.50359	2.45023	-0.55323	0	6882.328	
MTOR	P42345	10.04	-1.78854	0.765697	-0.94282	12.37024	17900.16	
MUS81	NA	19.1	-1.96497	0.153139	-1.18395	1.384083	NA	
MYCN	NA	42.19	9.594476	100	78.16791	100	NA	
NRAS	P01111	11.09	-1.39065	1.378254	-1.23925	1.730104	35119.58	
PDGFA	NA	27.83	1.12459	87.90199	-0.40937	23.70242	NA	
PDGFD	NA	9.3	-1.24868	0.459418	1.237985	88.58131	NA	
PDGFRA	NA	2.23	-1.40712	0.765697	-1.28371	0.17301	NA	
PDGFRB	P09619	14.3	-1.96909	0	-1.52893	0	39835.47	
PIK3CA	P42336	4.2	-1.78194	0.306279	-1.74049	0	6940.213	
PLCG1	P19174	113.09	0.91246	85.14548	1.857955	97.40484	56108.01	
RIF1	Q5UIP0	9.04	-0.09304	44.71669	0.13187	57.43945	5792.122	
RNF43	NA	22.3	17.65785	100	6.181564	100	NA	
ROS1	NA	8.35	552.9801	100	-0.07946	46.19377	NA	
RPTOR	Q8N122	10.21	0.044412	51.83767	-0.07809	46.02076	10613.26	
SF3B1	O75533	65.08	-2.19636	0.306279	-1.94784	0	242059.1	
SHMT1	P34896	10.08	-1.67518	0	-0.25914	36.24567	38350.82	
SMO	NA	21.31	0.085878	54.51761	2.110113	96.53979	NA	
SPINK1	NA	0	-0.41946	8.728943	-0.5928	1.730104	NA	
TP53	NA	34.2	2.125731	98.62175	1.89318	96.7128	NA	
VEGFA	NA	67.96	-1.76708	0.153139	-0.79491	3.114187	NA	
VEGFD	NA	1.41	-0.58174	11.02603	-0.76594	0	NA	
WNT3A	P56704	32.36	474.0645	100	3.780786	99.65398	7262.519	
YAP1	P46937	45.56	-1.21602	3.828484	-1.18651	3.287197	46057.27	
ZNRF3	NA	14.46	3.285072	99.84686	3.023957	98.78893	NA	

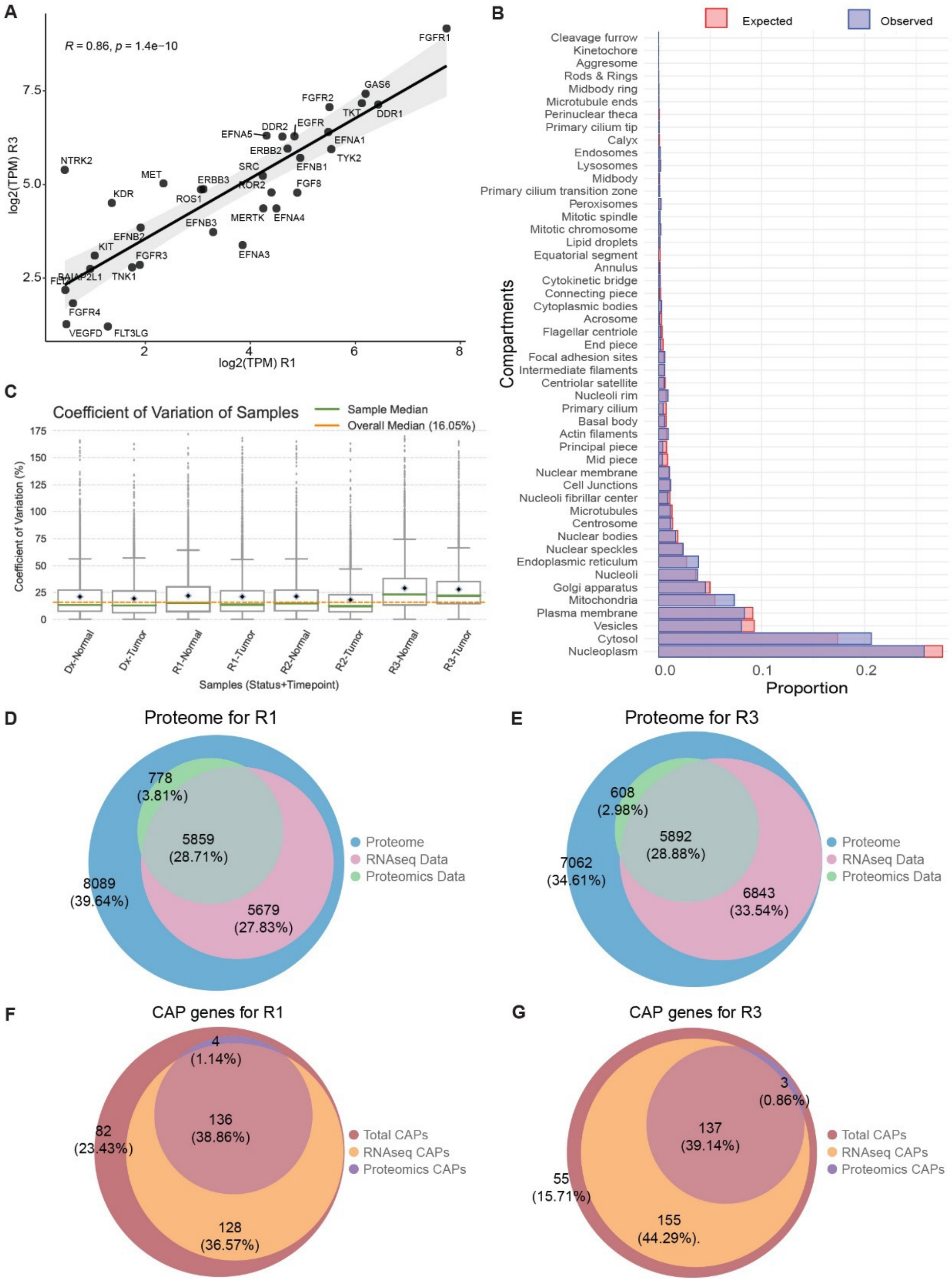

Appendix Figure S1: Proteogenomics of R1 and R3

A. Correlation plot of receptor tyrosine kinases from R1 and R3 relapses, with Pearson correlation coefficient r=0.86 and p value $1.4e^{-10}$. **B.** Evaluation of subcellular location annotations for quantified proteins showed the typical moderate enrichment of nucleoplasm, vesicular, and plasma membrane locations and enrichment of proteins annotated to be localized to the cytoplasm, mitochondria and endoplasmic reticulum with overall balanced coverage across all subcellular locations. Observed ratios were plotted against expected values in the Human

Protein Atlas. **C.** Distribution of protein quantification coefficients of variation across tissue samples collected at different time points. Box plots show the interquartile range (IQR, 25th to 75th percentile) with whiskers extending to 1.5×IQR. The median is indicated by a green line, the mean by a black diamond, and outliers are plotted as individual points beyond the whiskers. Data represents three technical replicates per group. **D-E.** Venn diagram showing the intersection of RNAseq and proteomics data of R1 (**D**) and R3 (**E**). **F-G.** Venn diagram showing the intersection of pediatric cancer-associated proteins (CAP) in the RNAseq and proteomics data of R1 (**F**) and R3 (**G**).

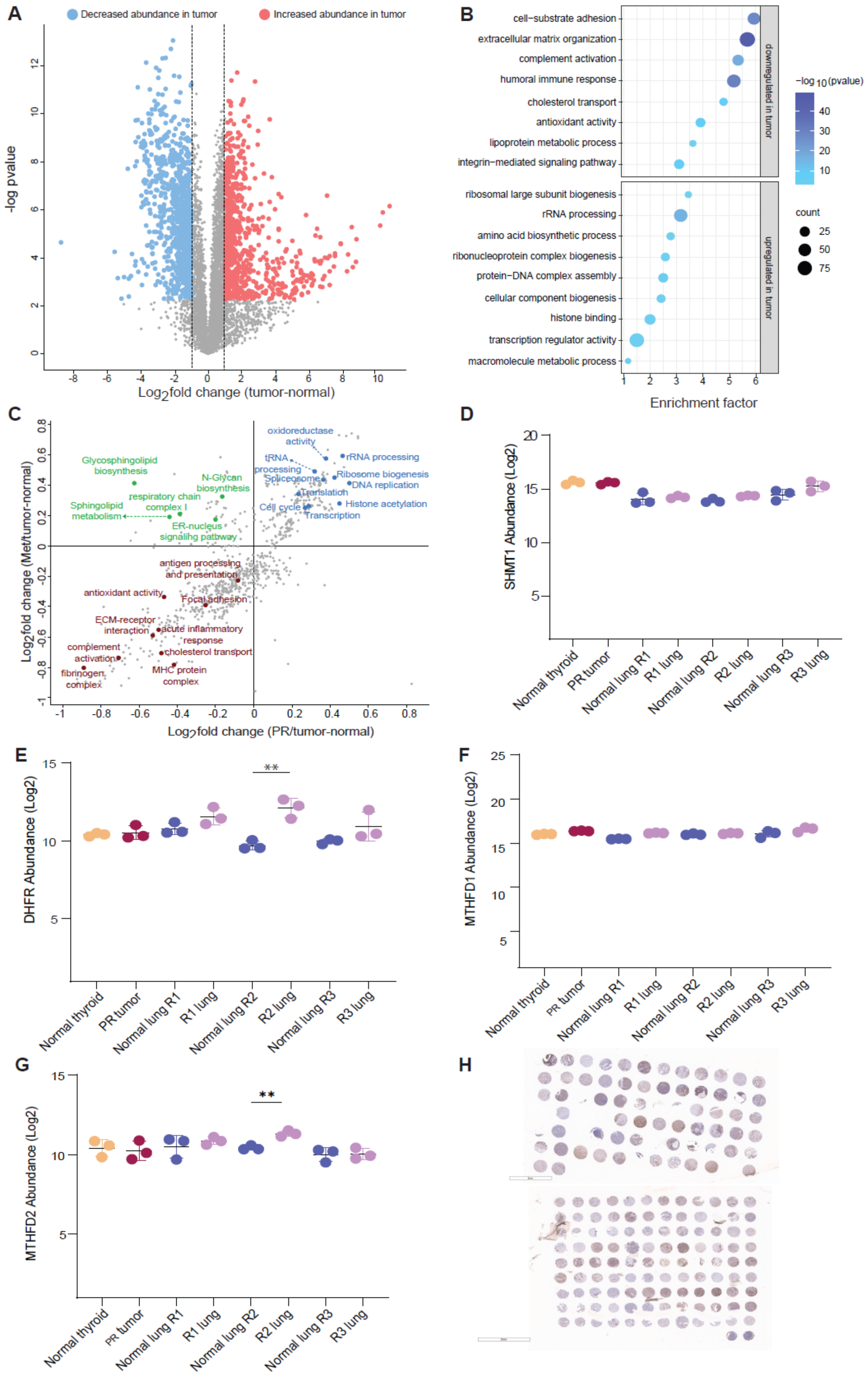
A
Decreased abundance in tumor
Increased abundance in tumor
-log pvalue
Log2fold change (tumor-normal)
B
cell−substrate adhesion
extracellular matrix organization
complement activation
humoral immune response
cholesterol transport
antioxidant activity
lipoprotein metabolic process
integrin−mediated signaling pathway
ribosomal large subunit biogenesis
rRNA processing
amino acid biosynthetic process
ribonucleoprotein complex biogenesis
protein−DNA complex assembly
cellular component biogenesis
histone binding
transcription regulator activity
macromolecule metabolic process
downregulated in tumor
upregulated in tumor
−log10(pvalue)
count
Enrichment factor
C
Glycosphingolipid biosynthesis
N-Glycan biosynthesis
respiratory chain complex I
Sphingolipid metabolism
ER-nucleus signaling pathway
oxidoreductase activity
tRNA processing
rRNA processing
Ribosome biogenesis
Spliceosome
DNA replication
Translation
Cell cycle
Histone acetylation
Transcription
antigen processing and presentation
antioxidant activity
Focal adhesion
ECM-receptor interaction
acute inflammatory response
complement activation
cholesterol transport
fibrinogen complex
MHC protein complex
Log2fold change (Met/tumor-normal)
Log2fold change (PR/tumor-normal)
D
SHMT1 Abundance (Log2)
Normal thyroid
PR tumor
Normal lung R1
R1 lung
Normal lung R2
R2 lung
Normal lung R3
R3 lung
E
DHFR Abundance (Log2)
**
F
MTHFD1 Abundance (Log2)
G
MTHFD2 Abundance (Log2)
**
H

Appendix Figure S2: Pathway analysis of the primary tumor and lung metastases

A. Volcano plot showing the global analysis of proteome perturbation between the tumor regions and adjacent normal regions from three technical replicates for each group. Proteins significant with student's t-test at 0.01 FDR and with log2 fold change >1 are highlighted in red for upregulation in tumor and in blue for downregulation in tumor. **B.** Pathway enrichment analysis of t-test significant proteins against GO terms using Fisher-exact test, FDR 0.05% show enrichment of proteins involved in transcription regulation, histone binding, protein-DNA complex assembly, amino acid biosynthesis in the primary and lung metastases. Proteins involved in extracellular matrix assembly, cell-substrate adhesion, cholesterol transport, antioxidant activity, and complement activation pathways were enriched in proteins with low abundance in the tumors. **C.** 2D-enrichment analyses of log2 fold changes between primary resection vs adjacent normal tissue and those between lung relapses vs corresponding adjacent normal tissues against GO terms using Fisher-exact test (FDR 0.05%). **D-G.** Abundance of 1C metabolism protein SHMT1 (**D**), DHFR (**E**), and Methylenetetrahydrofolate dehydrogenases MTHFD1 **(F)** and MTHFD2 **(G)** in the primary tumor and in the lung relapses, data shown represent three technical replicates for each group (average ± s.d.). Bonferroni's multiple comparisons test, for DHFR: **adjusted p value: normal lung R2 vs R2 lung=0.0094, and for MTHFD2: ** adjusted p value: normal lung R2 vs R2 lung=0.0097. **H.** SHMT2 IHC in TMA 15-012 (top) and TMA 21-003 (bottom).

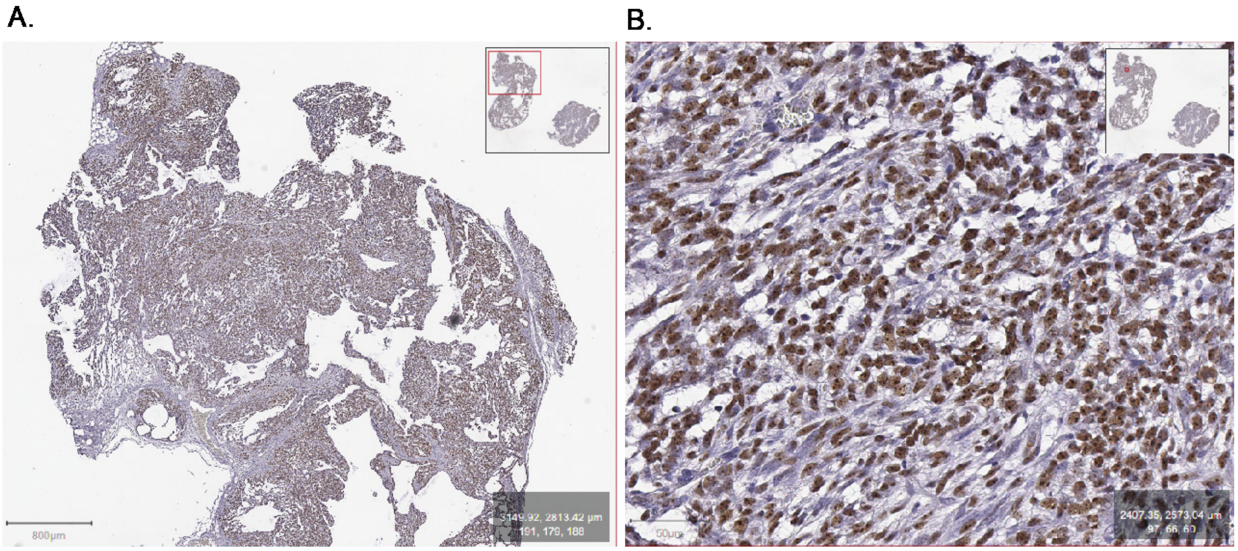

Appendix Figure S3: Characterization of SETTLE patient-derived mouse xenograft (NSG-PDX) model.

Shown are digitally scanned images of FFPE tissue sections isolated from a SETTLE NSG-PDX (*in vivo* passage 1) and stained with a human-specific nucleolin antibody (brown) confirming the presence of human tumor cells. Tissues were counterstained with Toludine blue (blue). Scale bar, 800 µm (left); 50 µm (right).

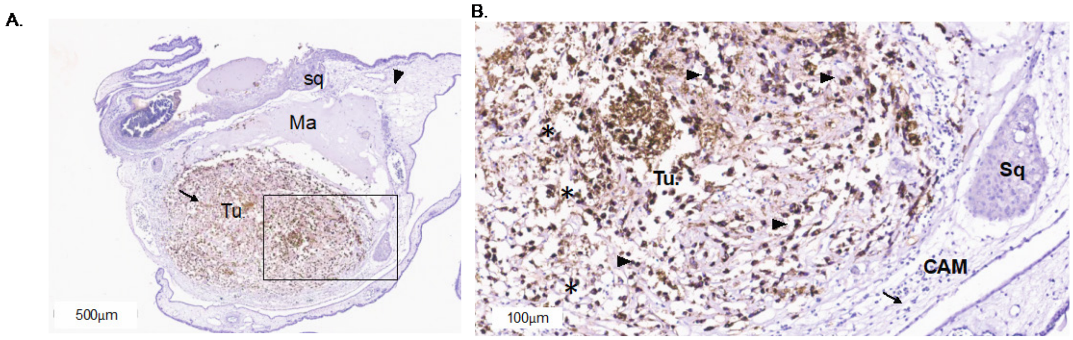

Appendix Figure S4: Characterization of SETTLE patient derived chorioallantoic membrane xenograft (CAM-PDX) model.

A. Low-power magnification of the CAM showing a cellular mass (Arrow) surrounded by the Matrigel (Ma). Squamous metaplasia (Sq) of the chorion is noted on the top of the CAM (arrowhead) and the tumor is encapsulated in the center (Tu.). The tumor in the center is well demarcated as indicated by IHC for a human-specific marker, LAMP1. **B.** Magnified image of CAM-PDX consists of largely discohesive cells with mild to moderate pleomorphism and hyperchromasia with some cells that are oval-to-spindled in appearance with irregular nuclear membranes and scant cytoplasm. Mitoses (Mi) are regularly identified**.** LAMP1 IHC shows strong cytoplasmic staining in tumor (tu. & arrowheads) cells but not in the CAM membrane (black arrow) or in the squamous metaplasia (sq).

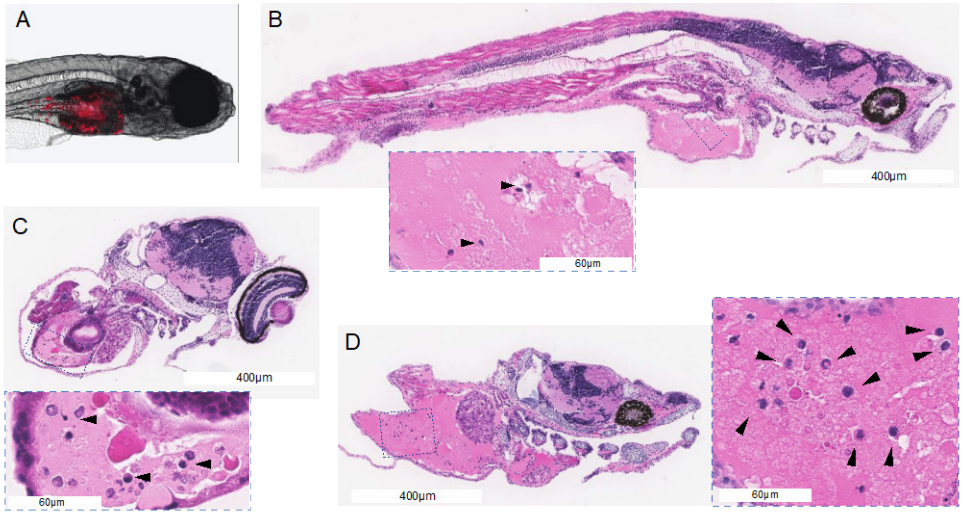

Appendix Figure S5: Characterization of zebrafish-PDX model of SETTLE.

A. Larval zebrafish xenografted with fluorescently labeled NSG-PDX-derived SETTLE tumor cells (red) at 4 days post-implantation (dpi) into the yolk sac. **B, C, D.** Representative H&E-stained FFPE sections of zebrafish-PDX showing SETTLE tumor cells within the yolk sac (arrowheads).

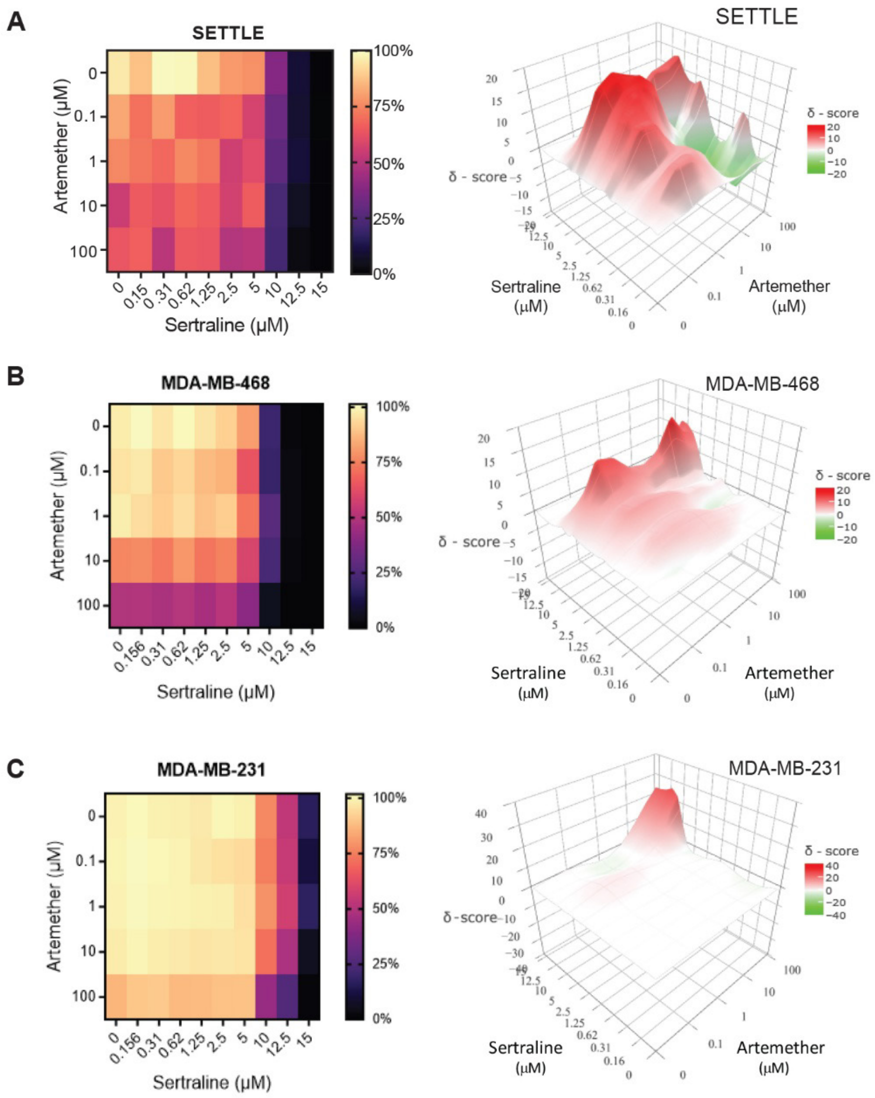

Appendix Figure S6: *In vitro* viability assays of SETTLE and breast cancer cell lines.
A-C. SETTLE (**A**), MDA-MB-468 (**B**) and MDA-MB 231 (**C**) subjected to treatment with sertraline and/or artemether shown as heat map viability plots (left) and the Highest Single Agent combinatorial drug synergy plots (d-score >10 indicative of synergistic effects) (right).
