# Supplementary figures and images for: Proteomics and personalized PDX models identify treatment for a progressive malignancy within an actionable timeframe
Source: EMBO Mol Med. 2025 Apr 1;17(4):625–44. doi: 10.1038/s44321-025-00212-8 (PMC11982353; doi:10.1038/s44321-025-00212-8)

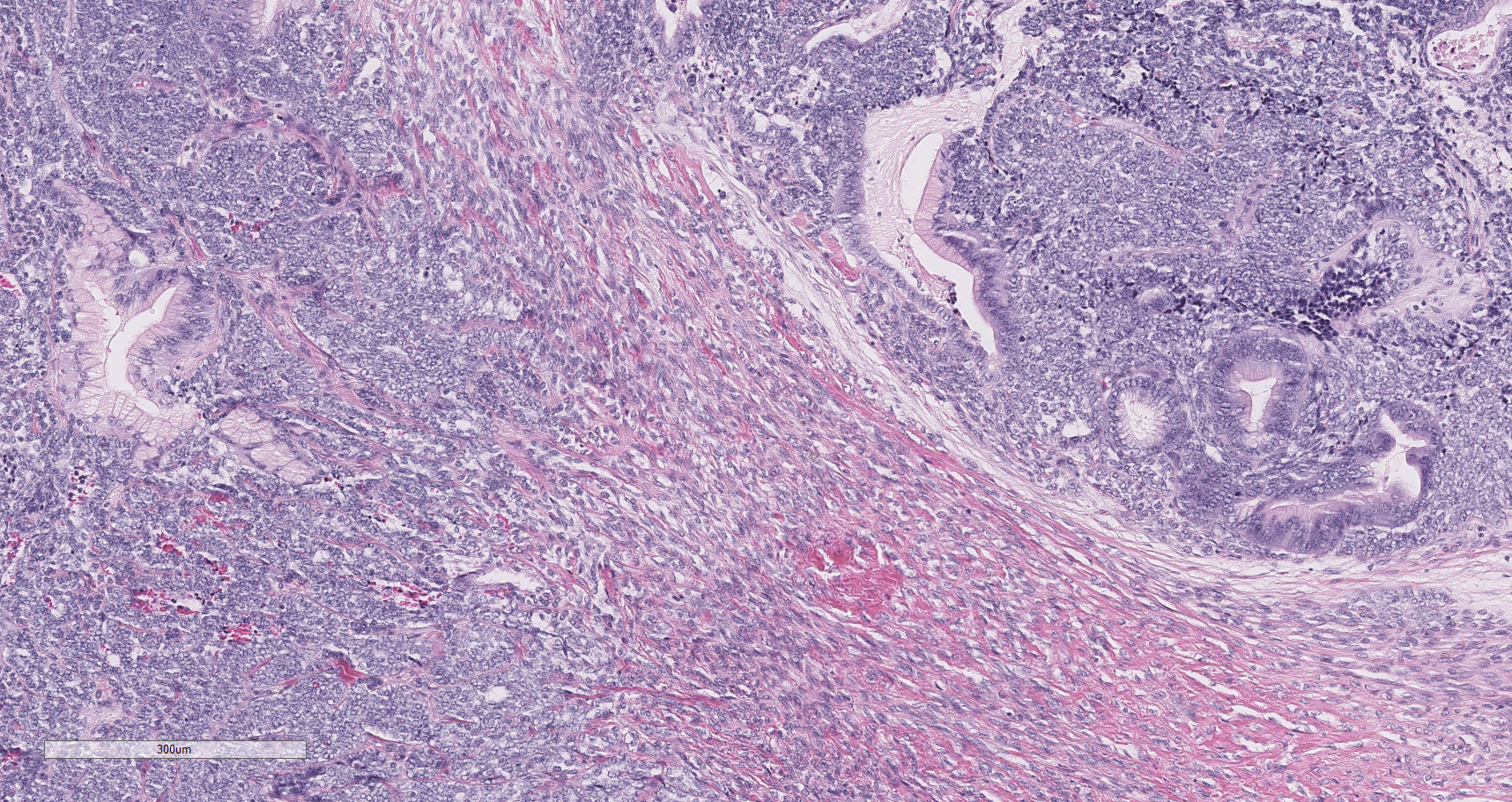

Supplement: Supplementary file 3 — Source data Fig. 1 [file 44321_2025_212_MOESM3_ESM.zip › Figure 1/1C/Figure 1C_PR_HnE10x.tif]

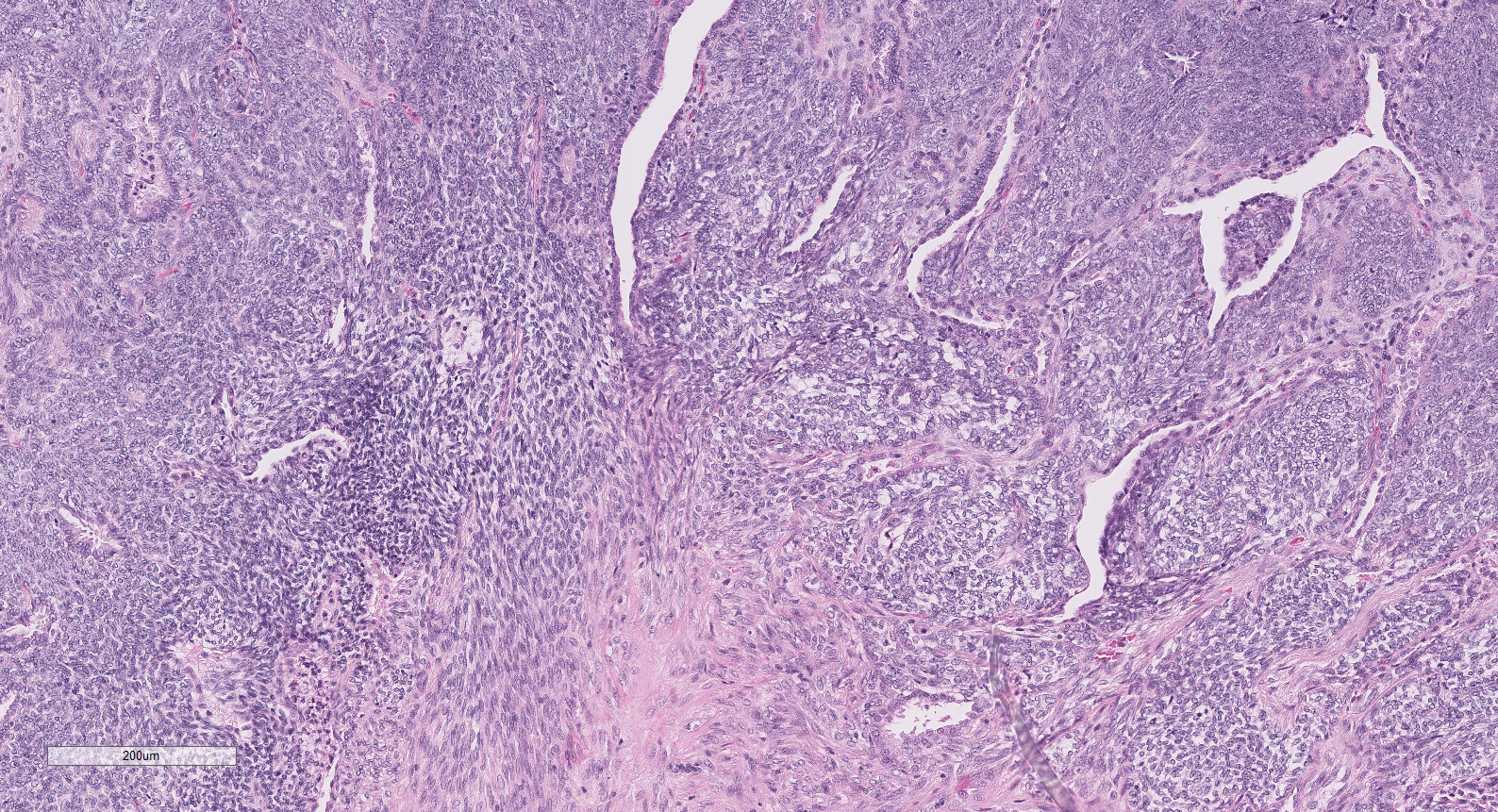

Supplement: Supplementary file 3 — Source data Fig. 1 [file 44321_2025_212_MOESM3_ESM.zip › Figure 1/1C/Figure 1C_R1_HnE10x.tif]

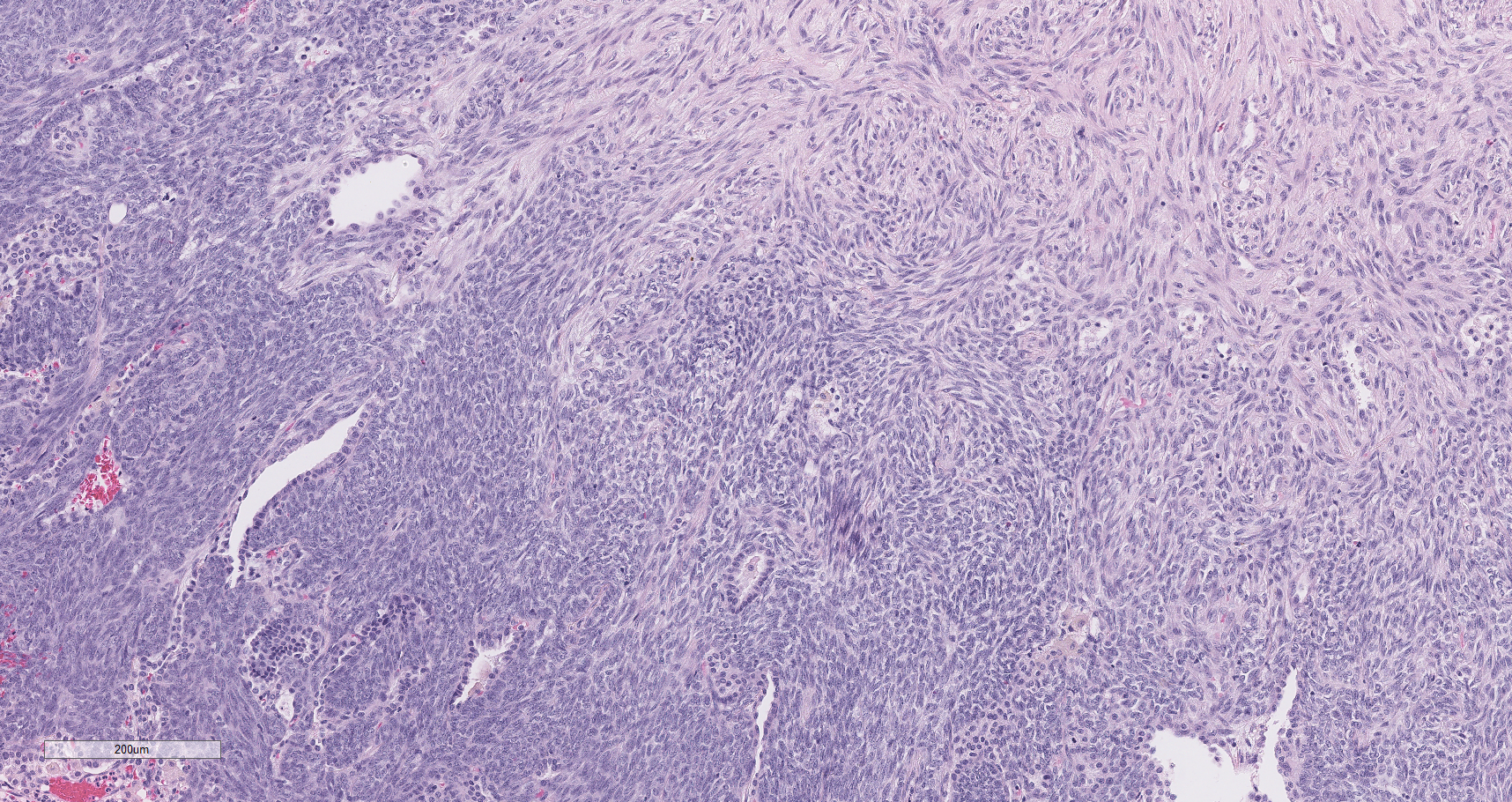

Supplement: Supplementary file 3 — Source data Fig. 1 [file 44321_2025_212_MOESM3_ESM.zip › Figure 1/1C/Figure 1C_R2_HnE10x.tif]

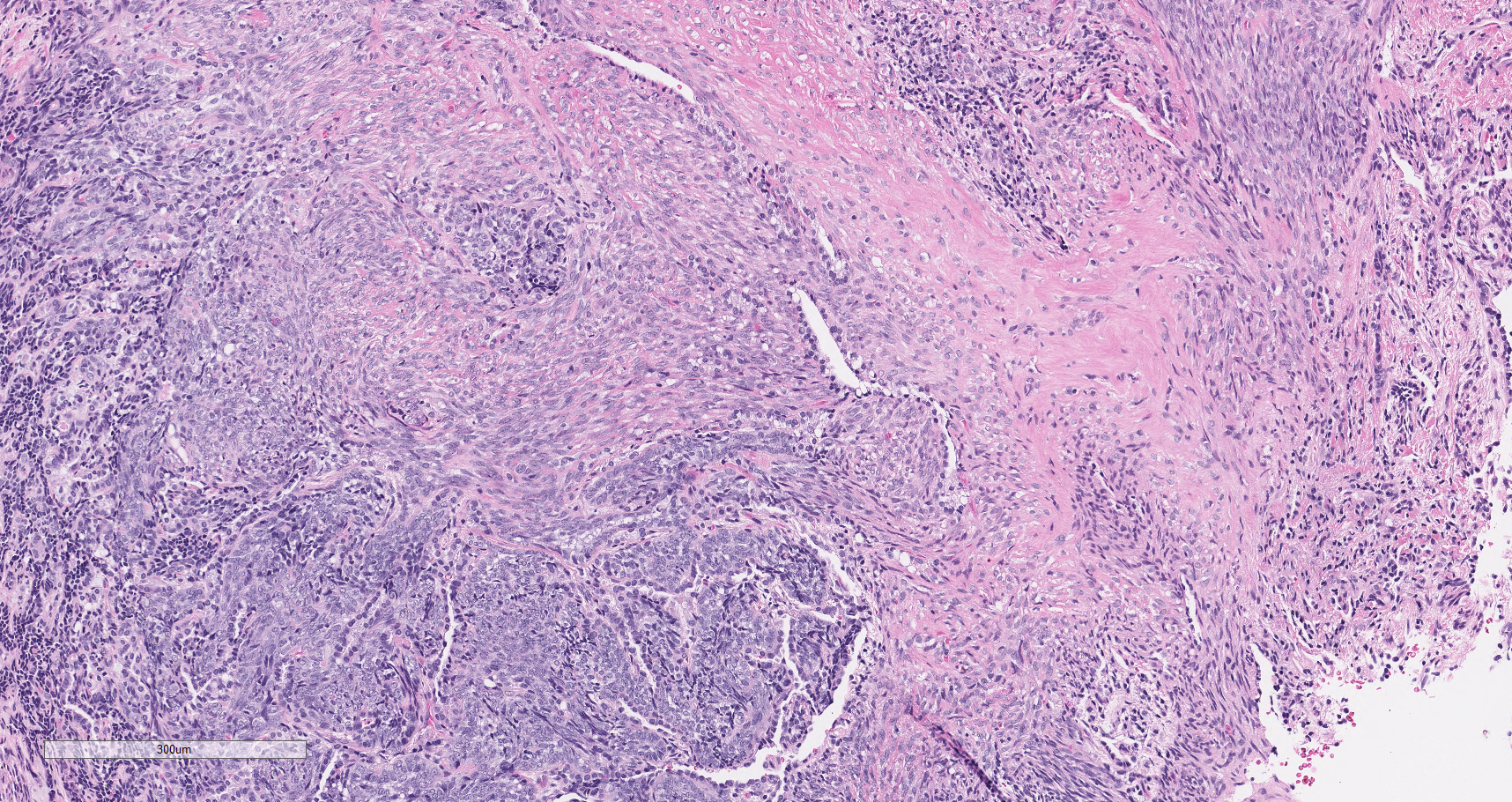

Supplement: Supplementary file 3 — Source data Fig. 1 [file 44321_2025_212_MOESM3_ESM.zip › Figure 1/1C/Figure 1C_R3_HnE10x.tif]

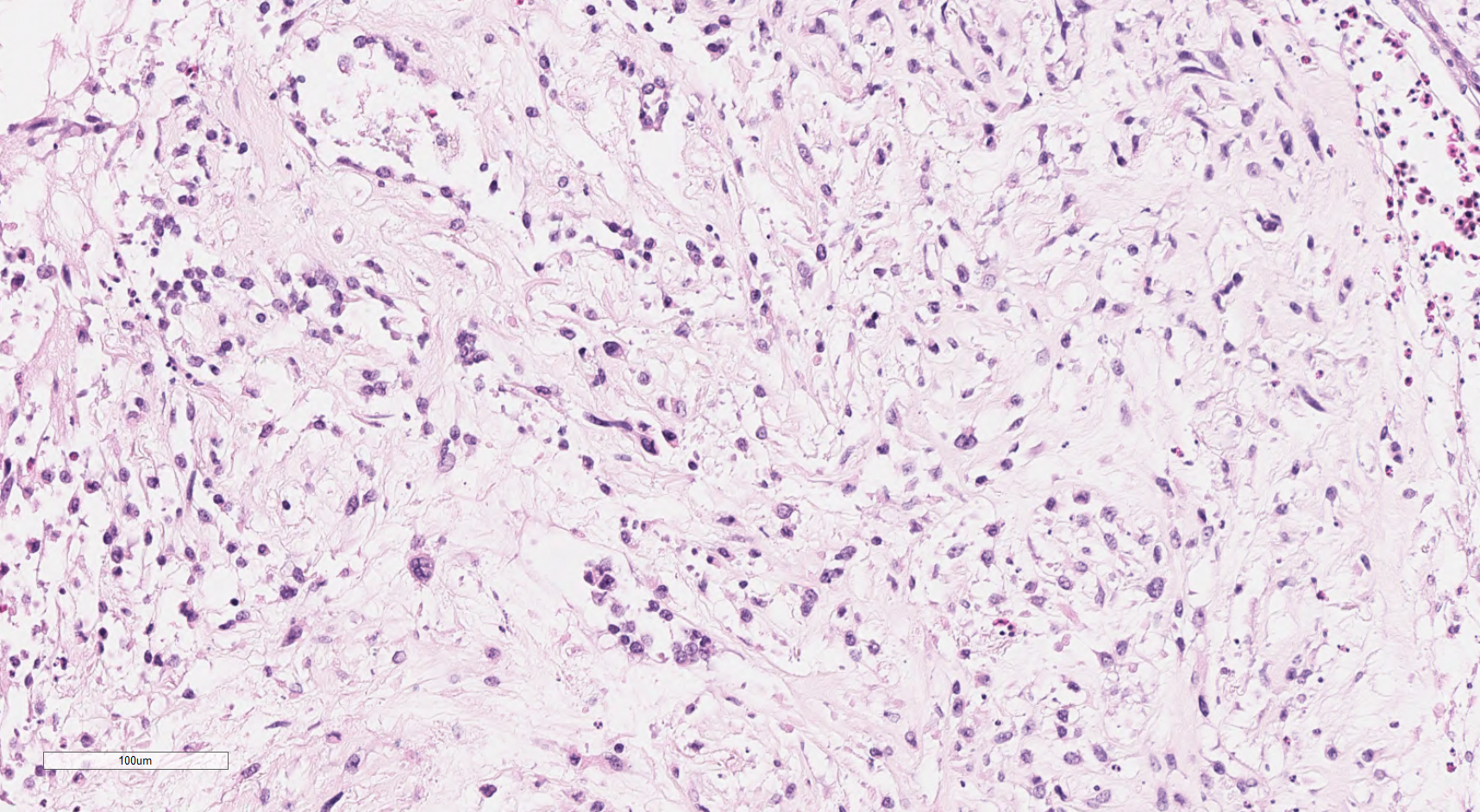

Supplement: Supplementary file 4 — Source data Fig. 2 [file 44321_2025_212_MOESM4_ESM.zip › Figure 2/2F/Figure 2 F_CAM-HnE.tif]

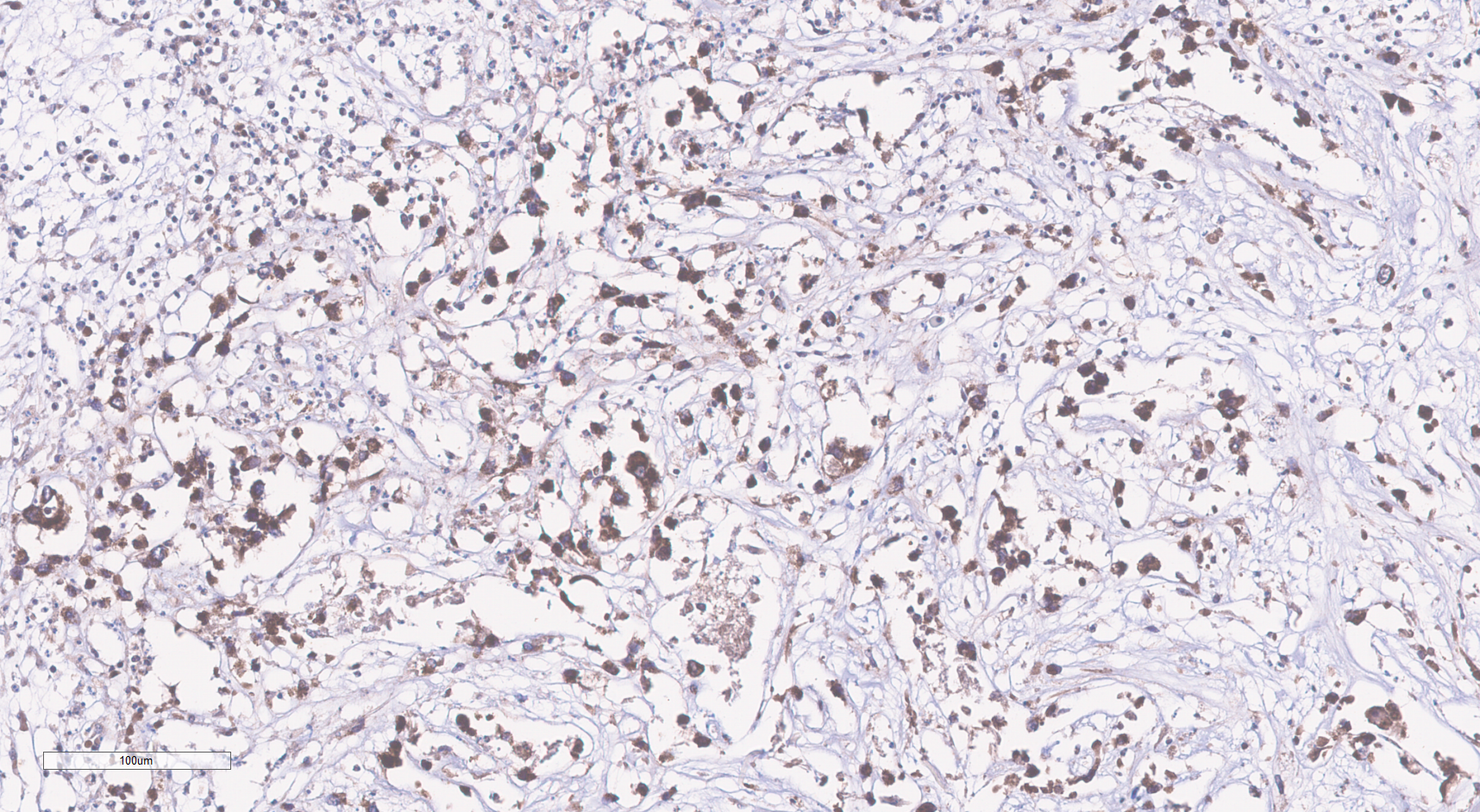

Supplement: Supplementary file 4 — Source data Fig. 2 [file 44321_2025_212_MOESM4_ESM.zip › Figure 2/2F/Figure 2 F_CAM-SHMT2.tif]

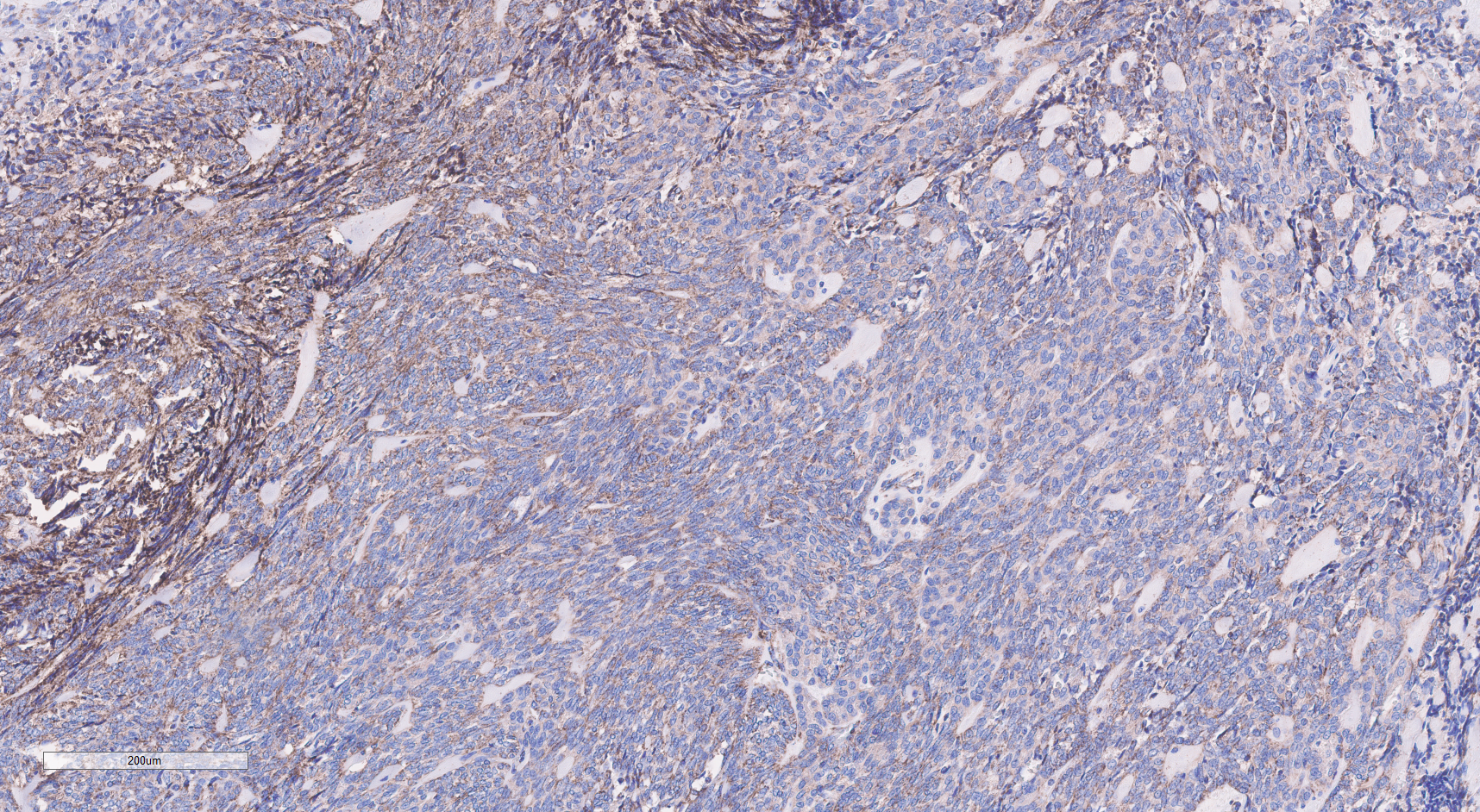

Supplement: Supplementary file 4 — Source data Fig. 2 [file 44321_2025_212_MOESM4_ESM.zip › Figure 2/2C/Figure 2 C-PR-shmt2.tif]

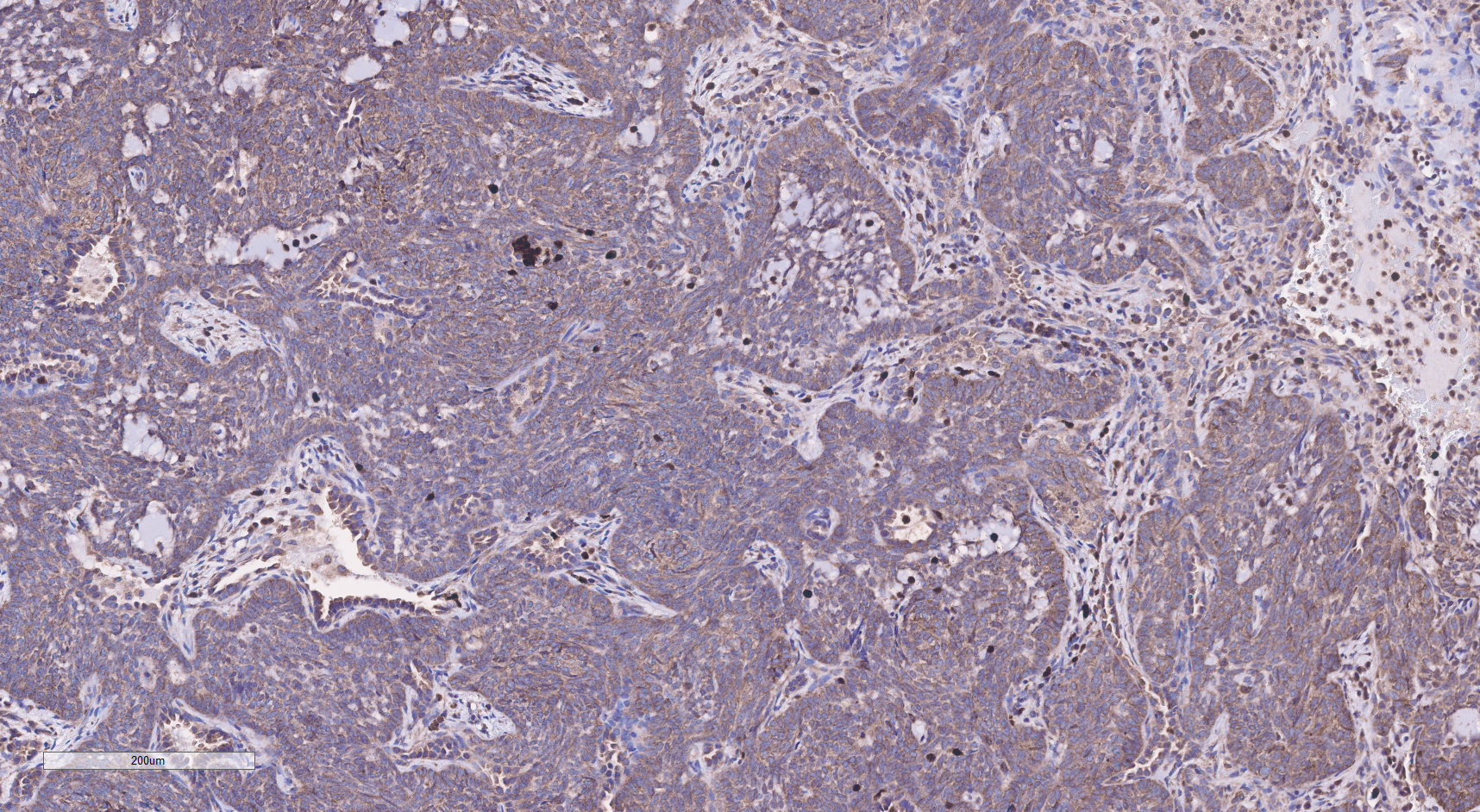

Supplement: Supplementary file 4 — Source data Fig. 2 [file 44321_2025_212_MOESM4_ESM.zip › Figure 2/2C/Figure 2 C-R1-shmt2.tif]

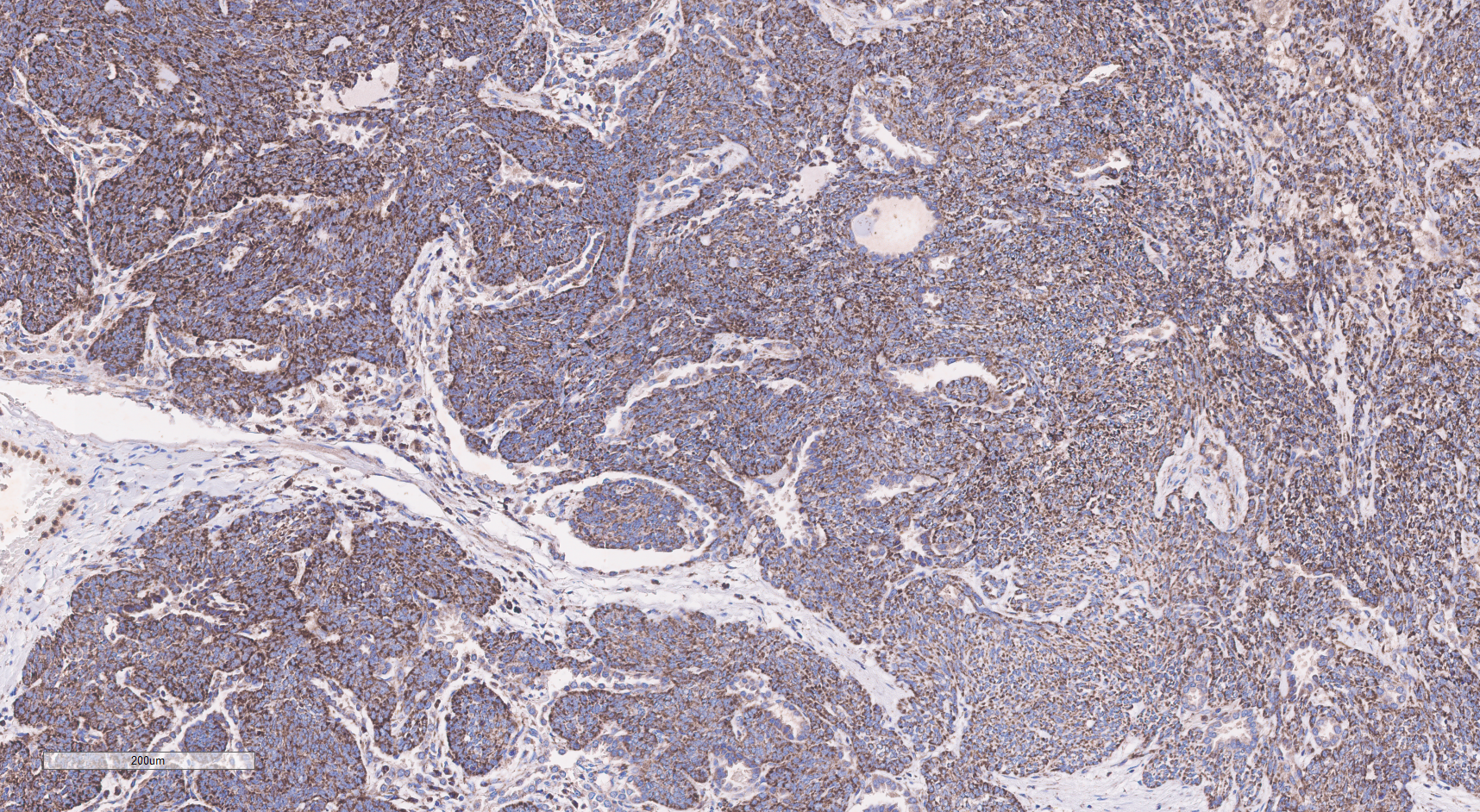

Supplement: Supplementary file 4 — Source data Fig. 2 [file 44321_2025_212_MOESM4_ESM.zip › Figure 2/2C/Figure 2 C-R2-shmt2.tif]

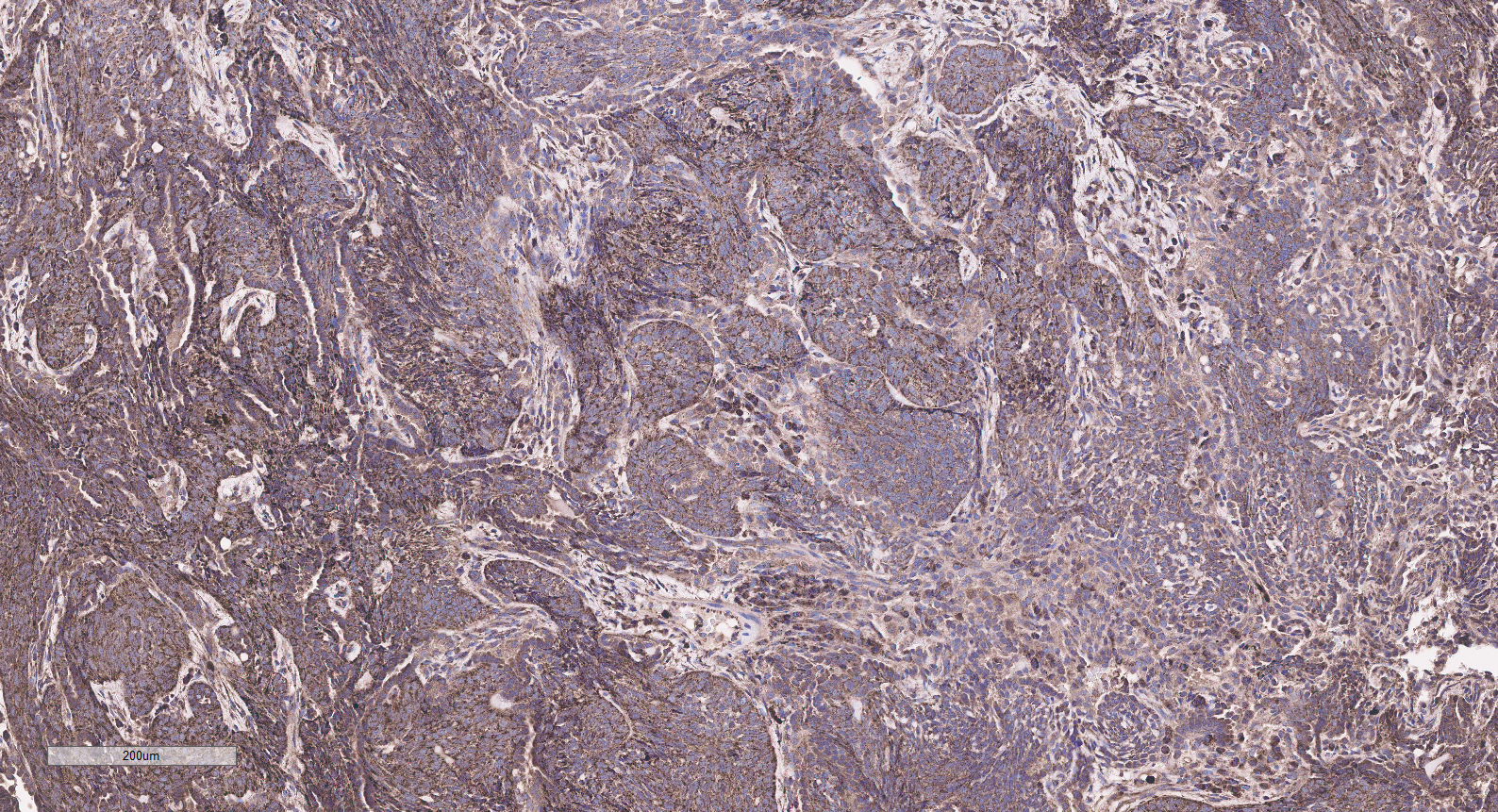

Supplement: Supplementary file 4 — Source data Fig. 2 [file 44321_2025_212_MOESM4_ESM.zip › Figure 2/2C/Figure 2 C-R3-shmt2.tif]

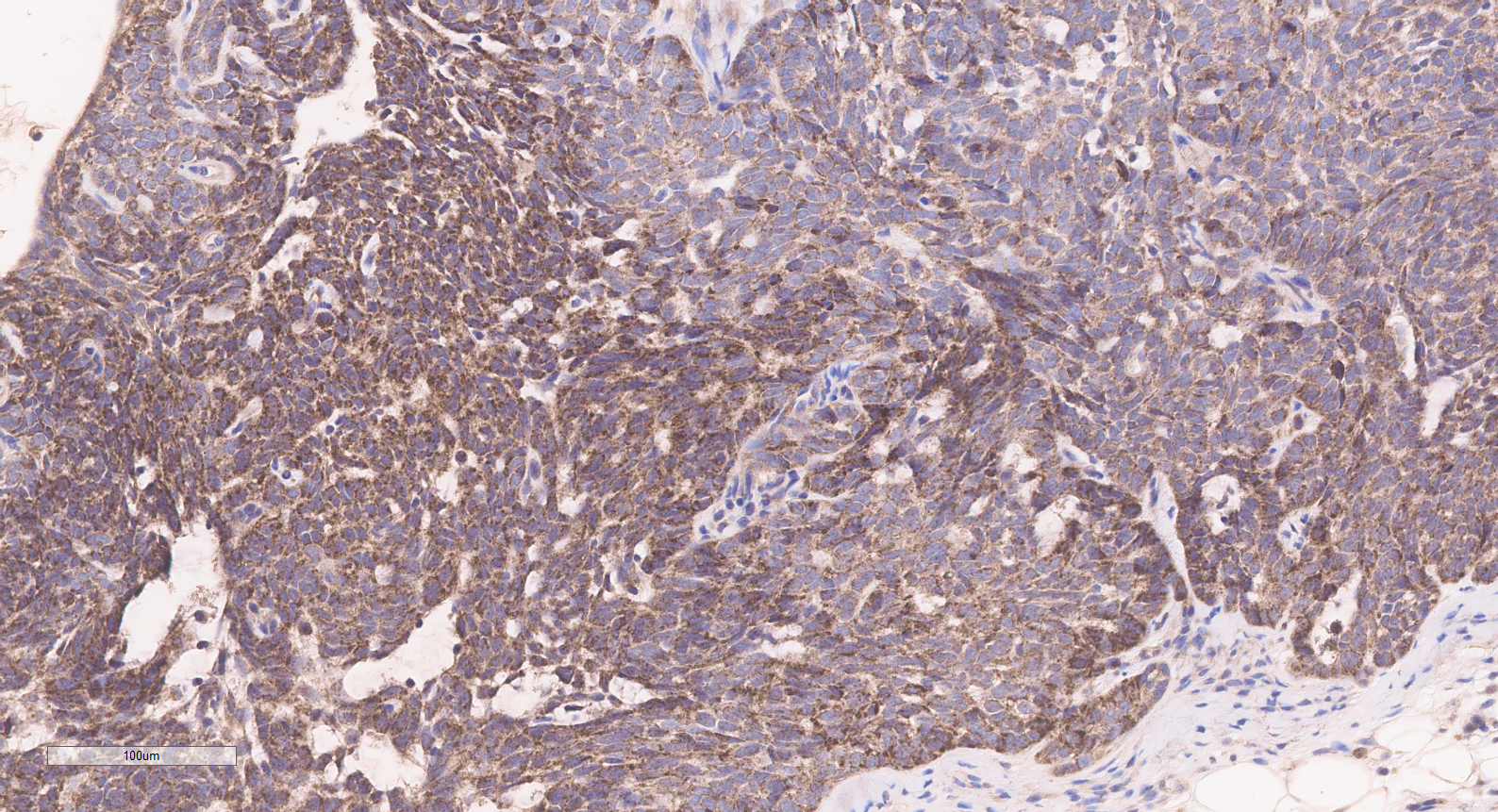

Supplement: Supplementary file 4 — Source data Fig. 2 [file 44321_2025_212_MOESM4_ESM.zip › Figure 2/2E/Figure 2 E_Mouse_SHMT2-settlePDX.tif]

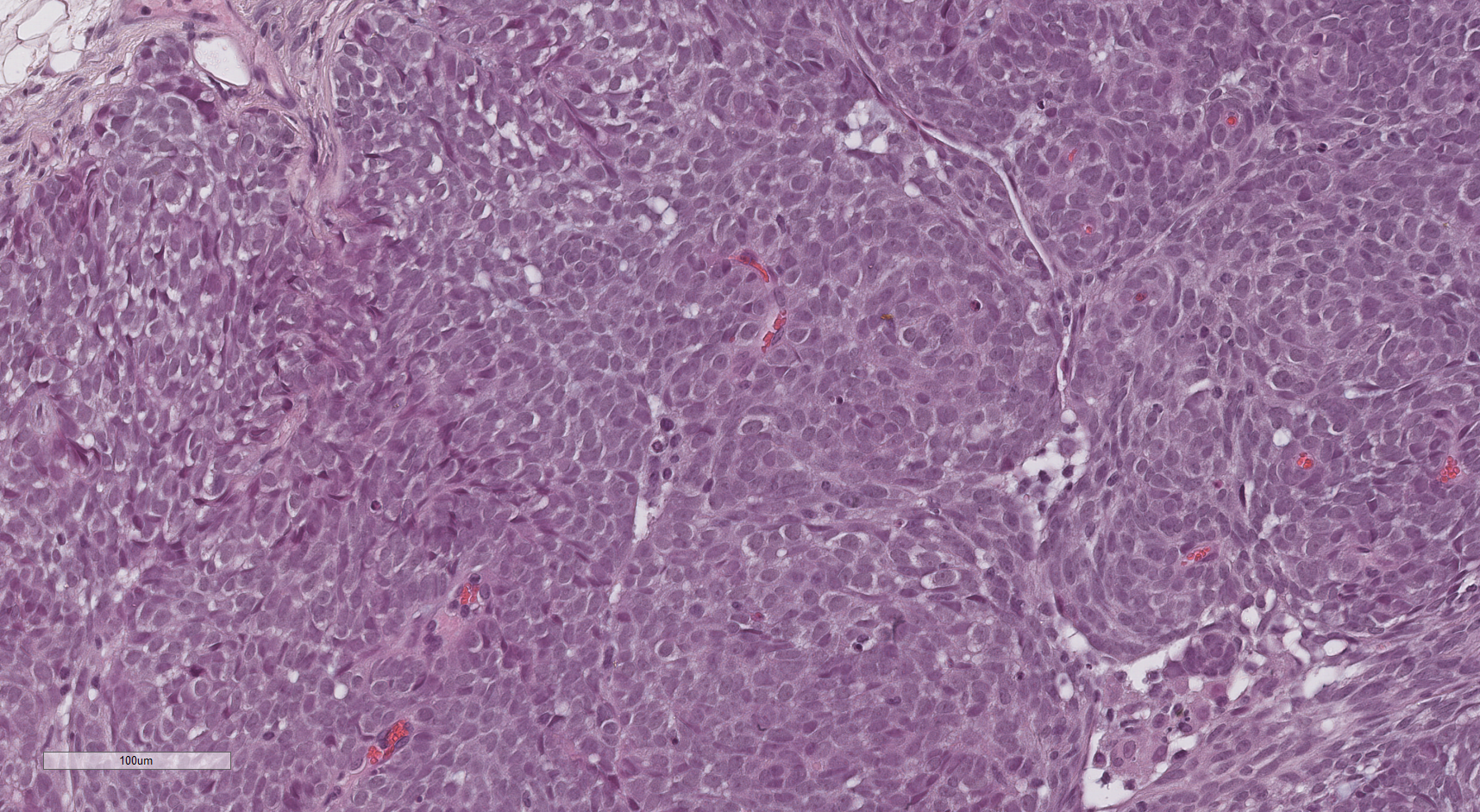

Supplement: Supplementary file 4 — Source data Fig. 2 [file 44321_2025_212_MOESM4_ESM.zip › Figure 2/2E/Figure 2 E_Mouse_HnE-settlePDX.tif]

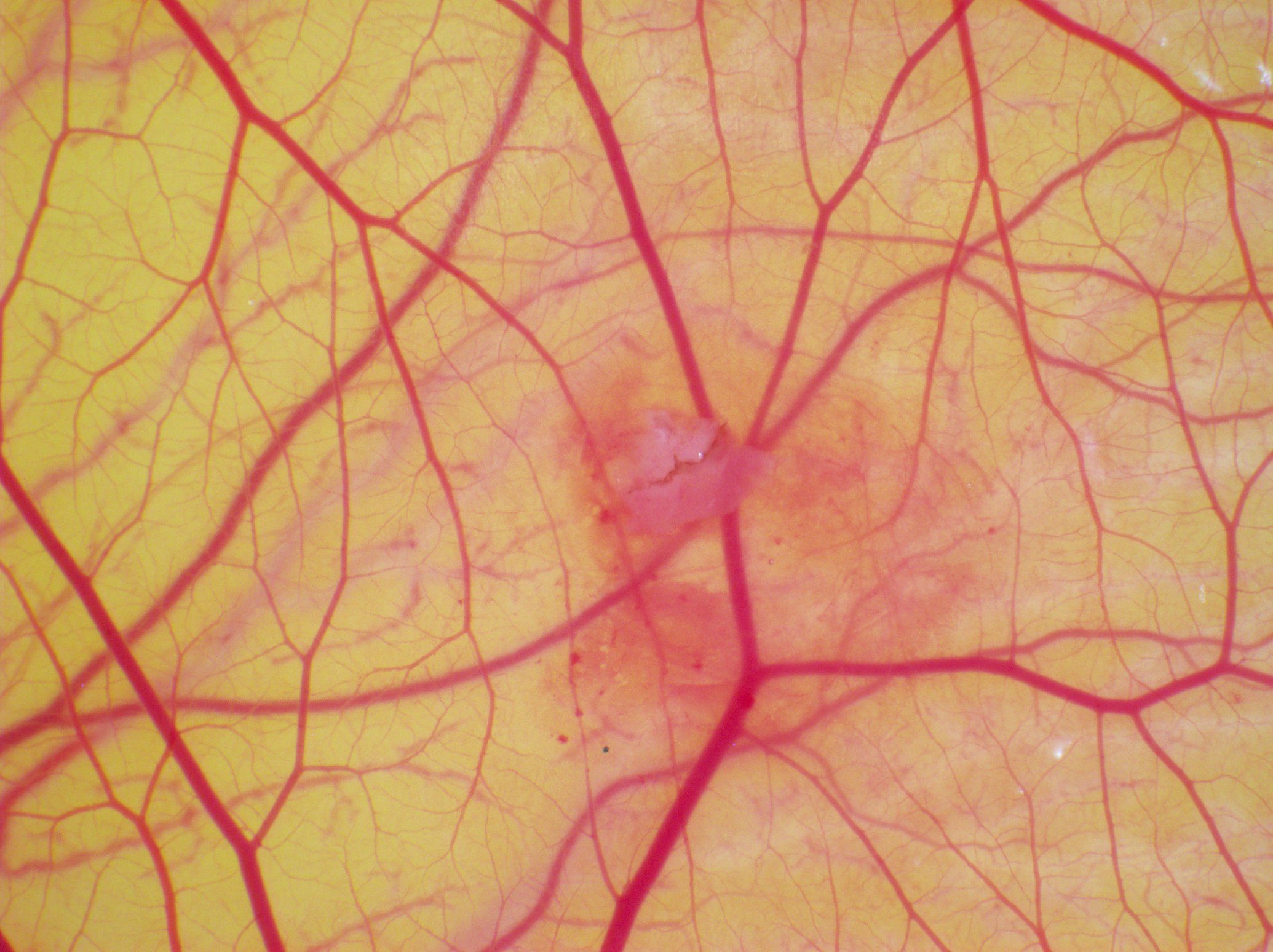

Supplement: Supplementary file 5 — Source data Fig. 3 [file 44321_2025_212_MOESM5_ESM.zip › Figure 3/3A/Figure 3 A_D1-SRT-2mg.jpg]

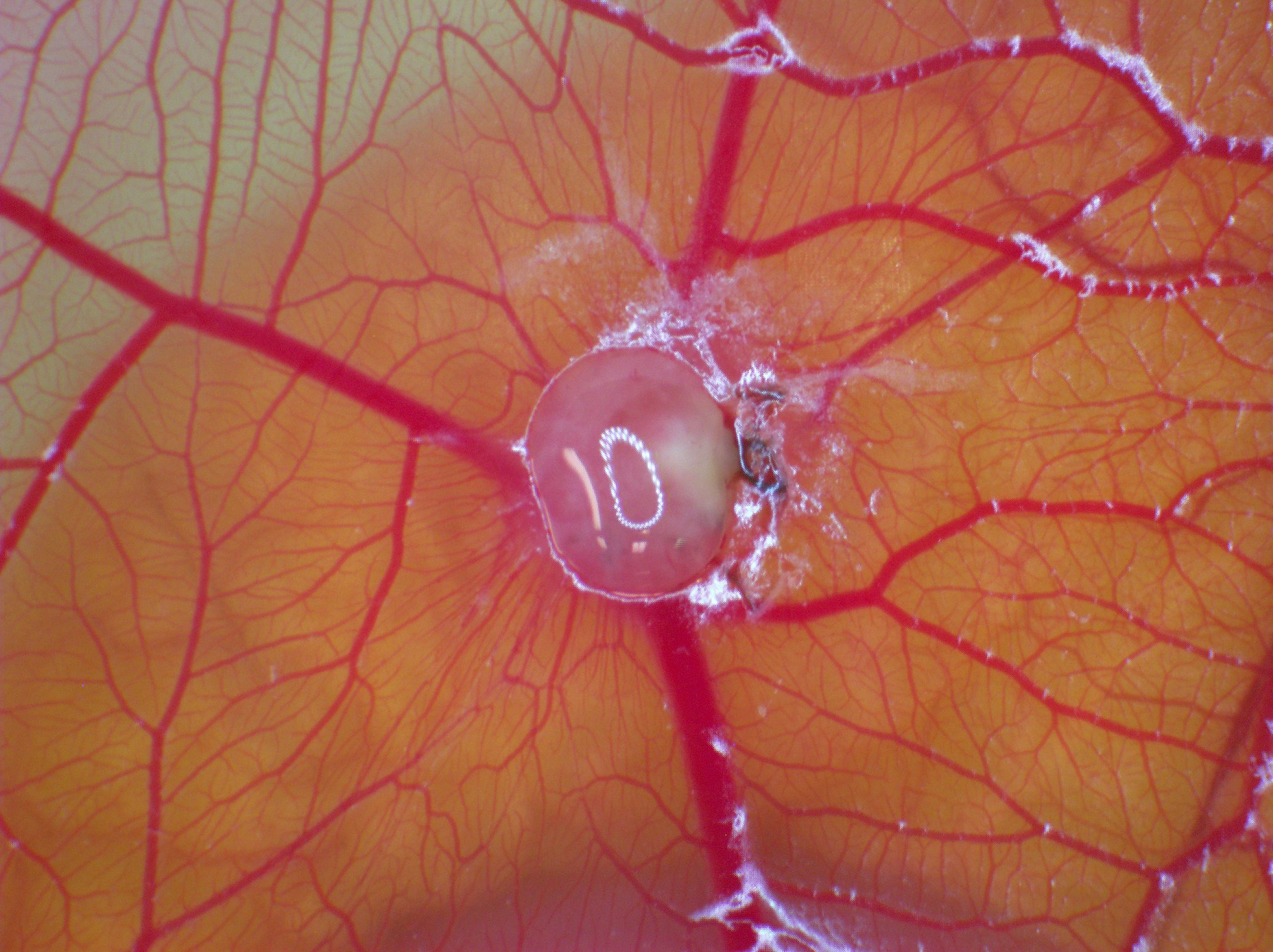

Supplement: Supplementary file 5 — Source data Fig. 3 [file 44321_2025_212_MOESM5_ESM.zip › Figure 3/3A/Figure 3 A_D5-Vehicle Control.jpg]

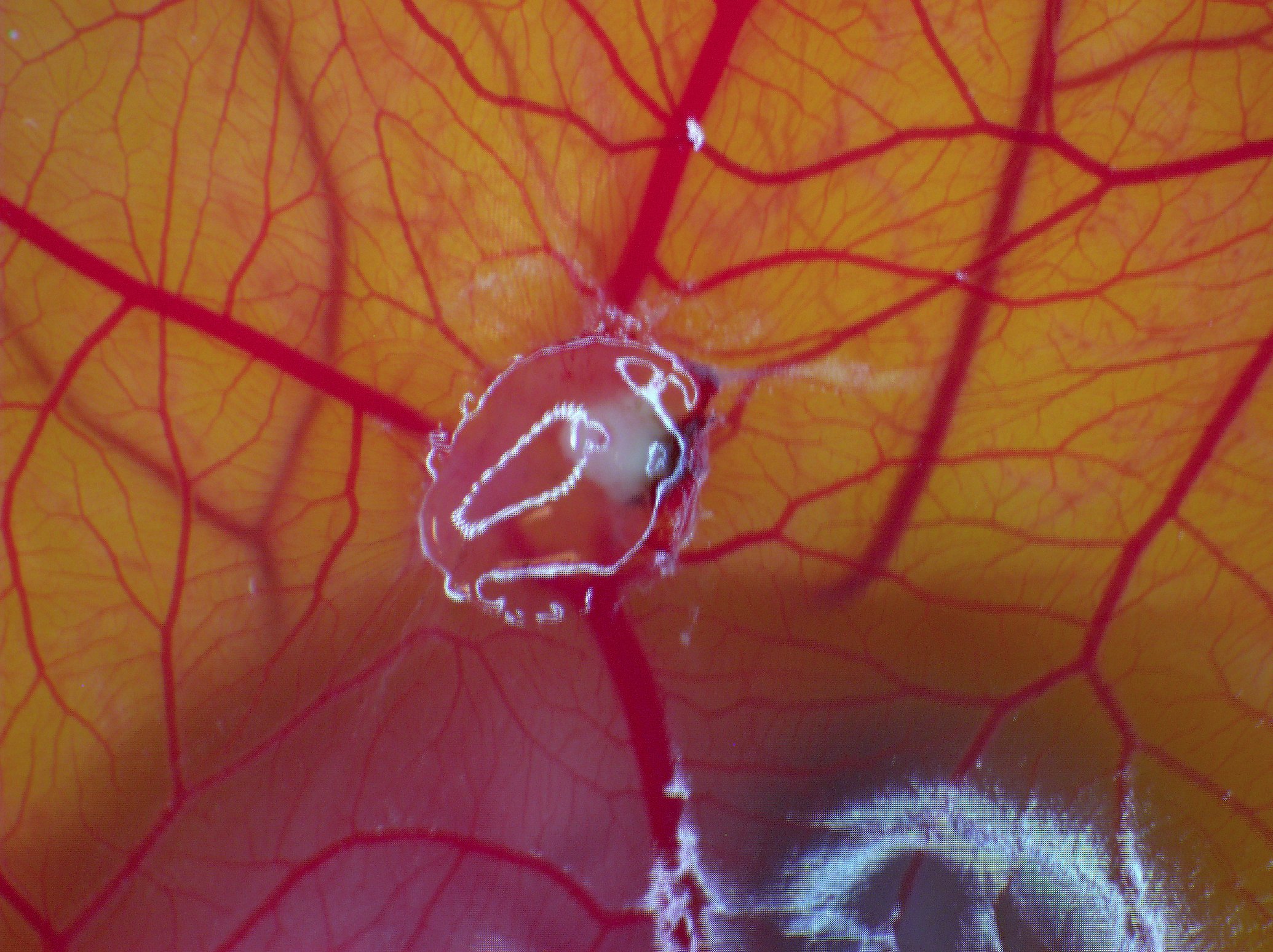

Supplement: Supplementary file 5 — Source data Fig. 3 [file 44321_2025_212_MOESM5_ESM.zip › Figure 3/3A/Figure 3 A_D3-Vehicle Control.jpg]

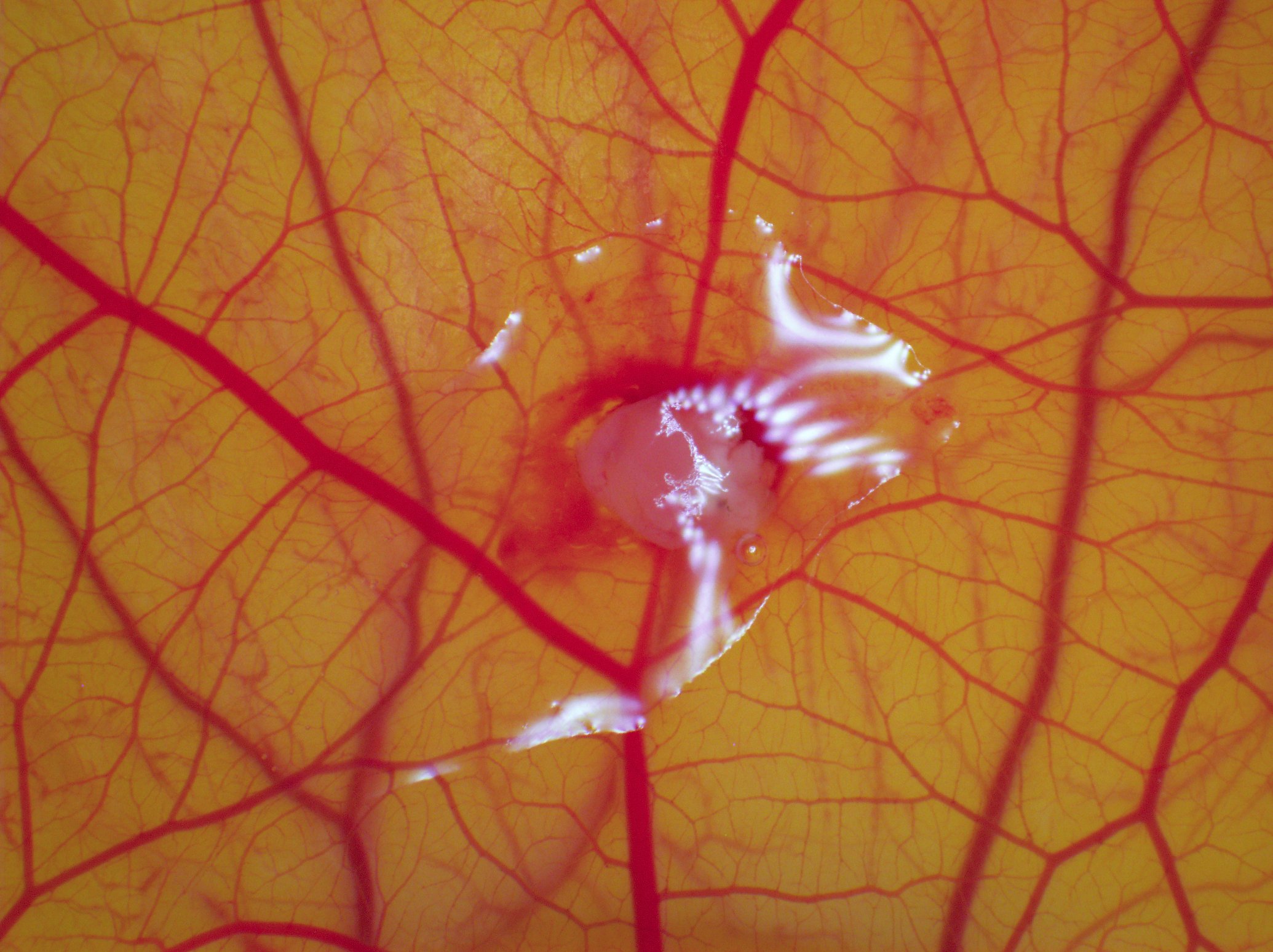

Supplement: Supplementary file 5 — Source data Fig. 3 [file 44321_2025_212_MOESM5_ESM.zip › Figure 3/3A/Figure 3 A_D1-Vehicle Control.jpg]

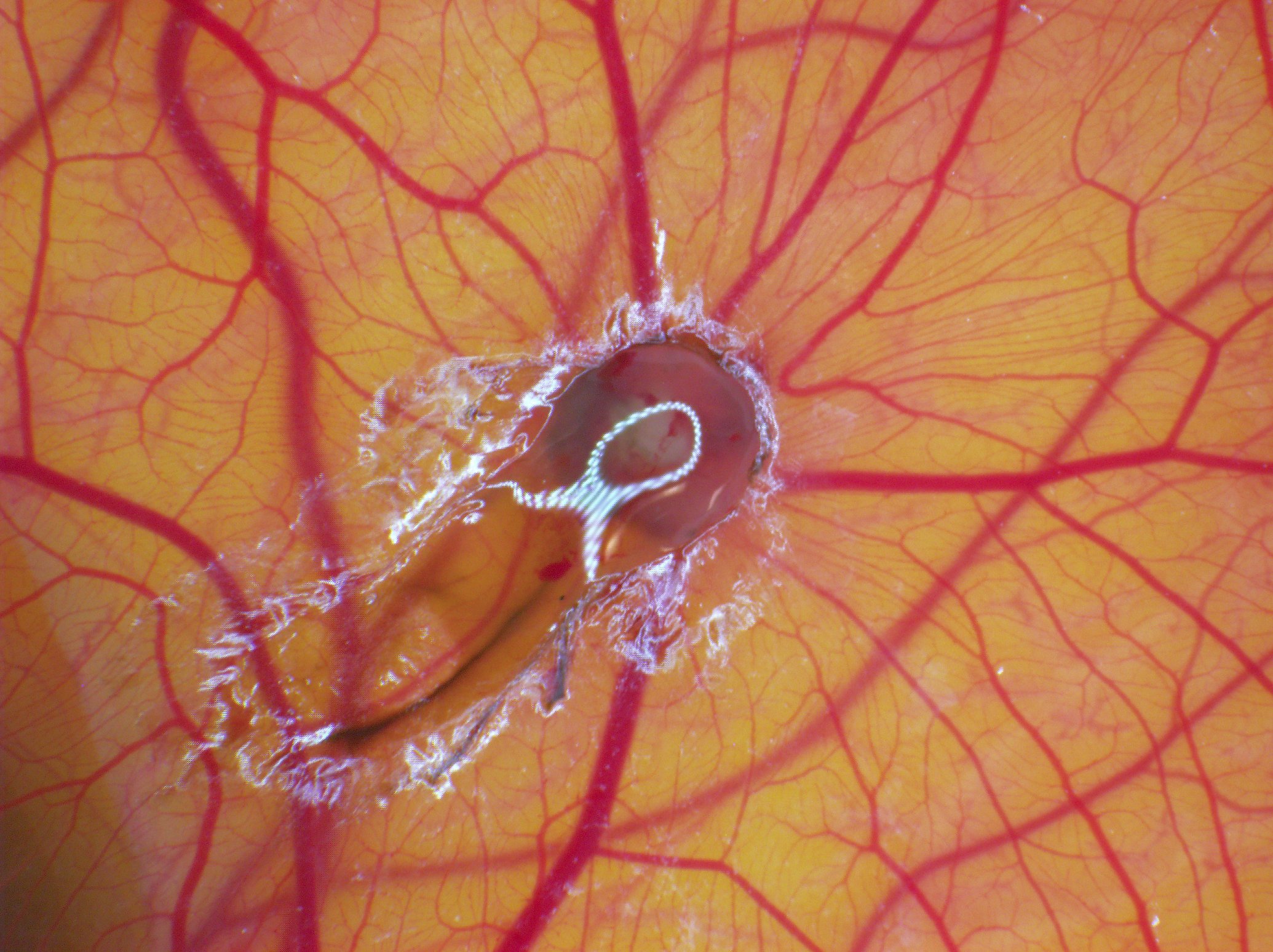

Supplement: Supplementary file 5 — Source data Fig. 3 [file 44321_2025_212_MOESM5_ESM.zip › Figure 3/3A/Figure 3 A_D3-SRT-2mg.jpg]

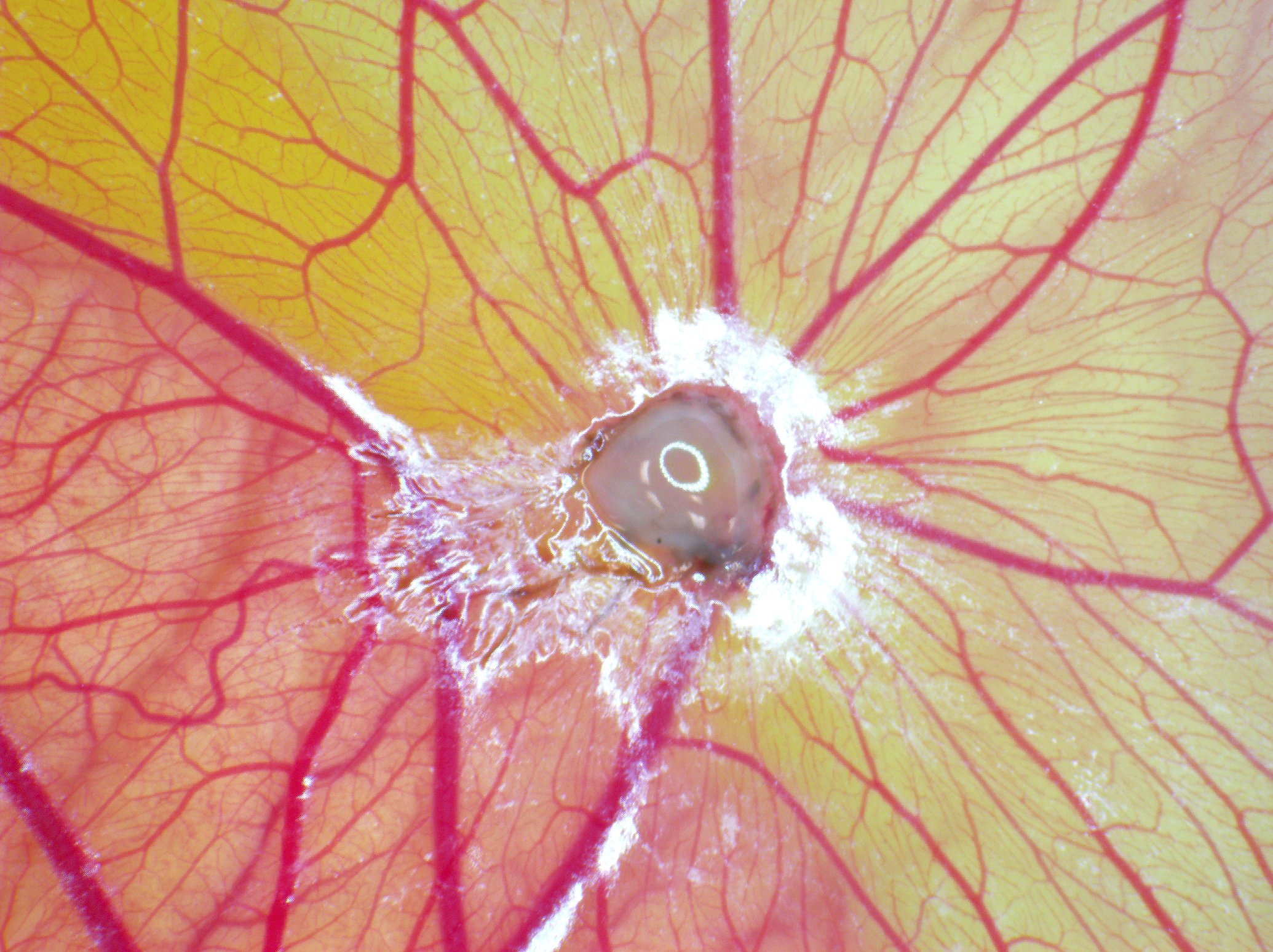

Supplement: Supplementary file 5 — Source data Fig. 3 [file 44321_2025_212_MOESM5_ESM.zip › Figure 3/3A/Figure 3 A_D5-SRT-2mg.jpg]

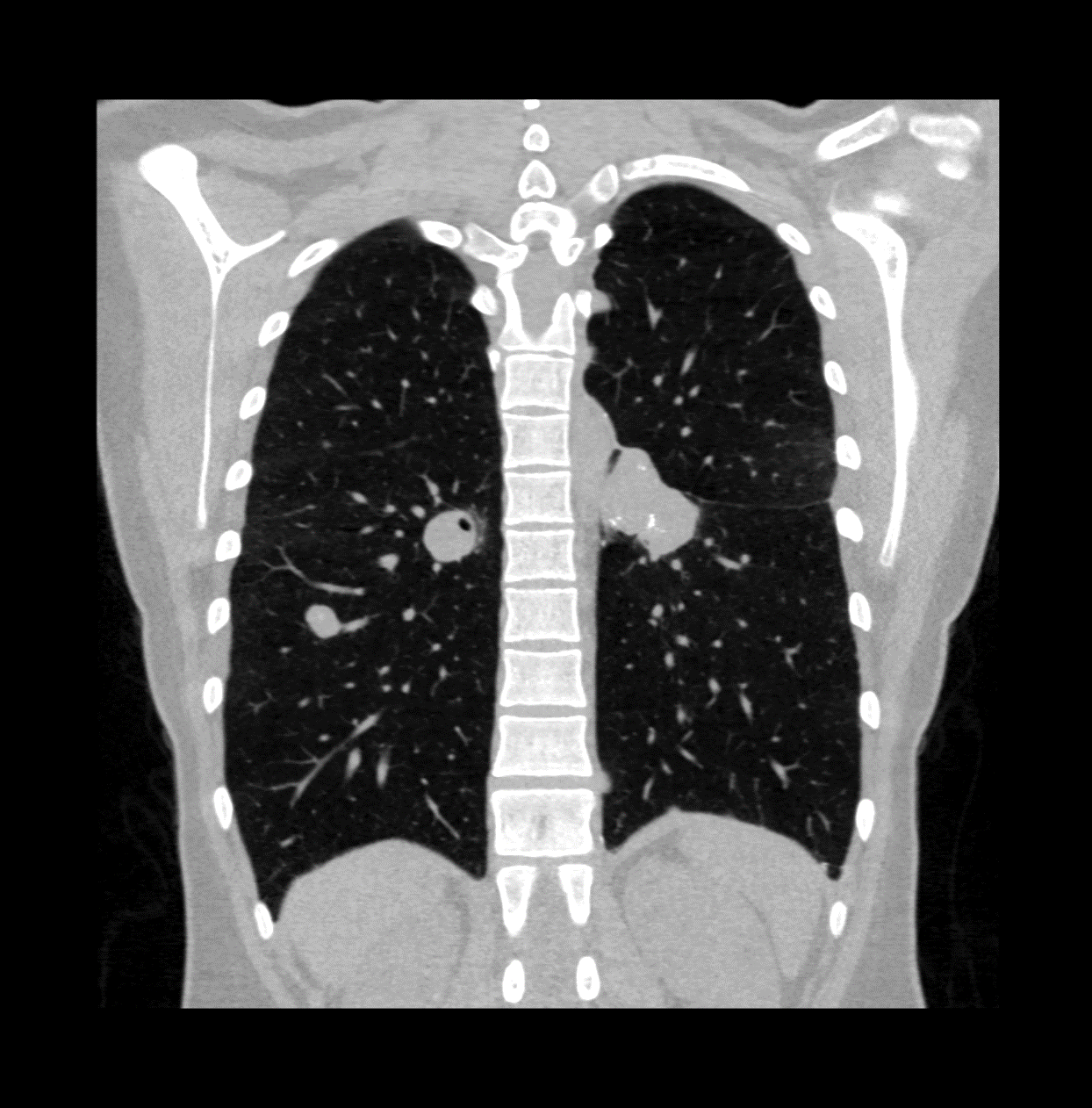

Supplement: Supplementary file 5 — Source data Fig. 3 [file 44321_2025_212_MOESM5_ESM.zip › Figure 3/3F/co-m2.png]

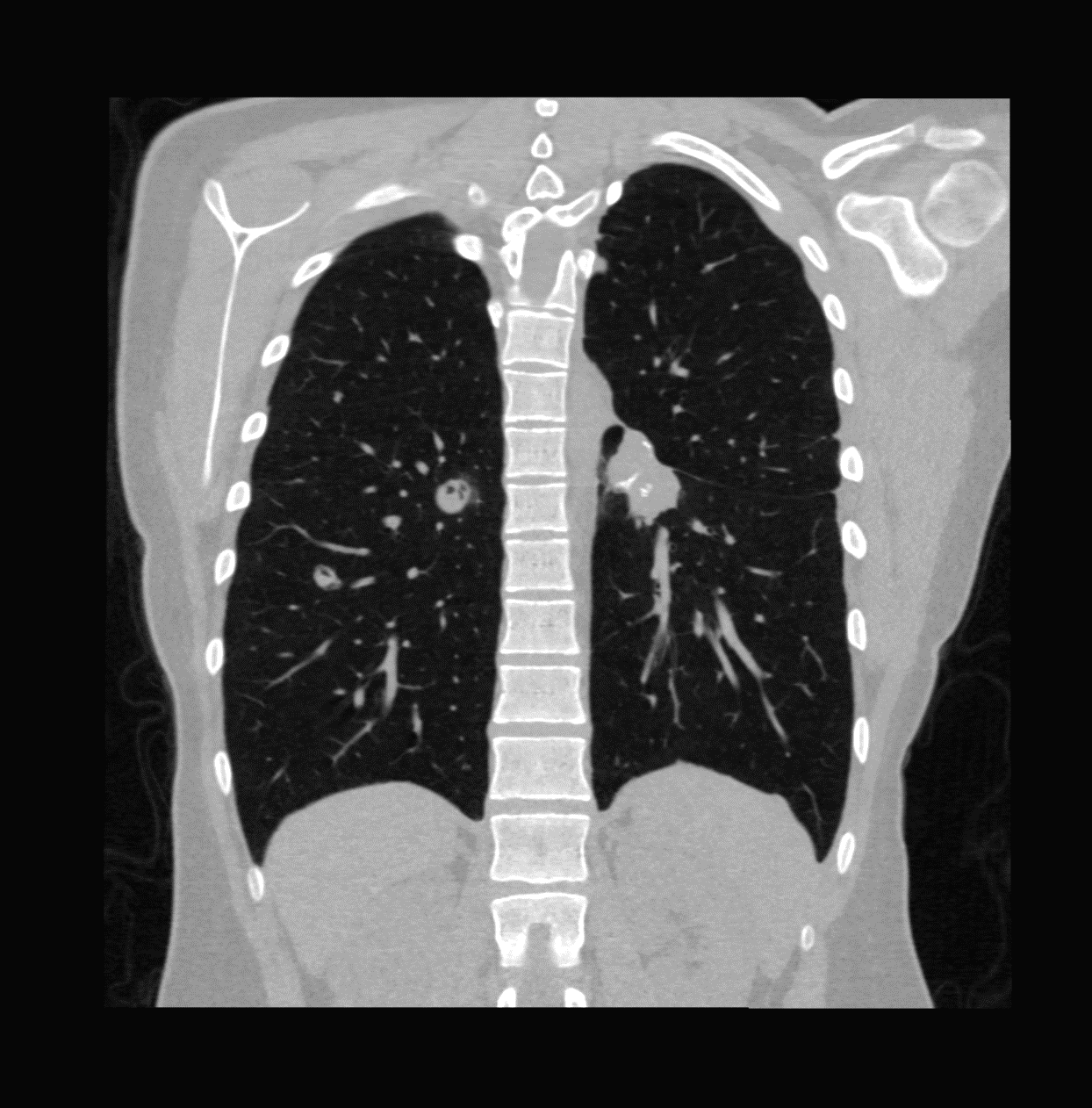

Supplement: Supplementary file 5 — Source data Fig. 3 [file 44321_2025_212_MOESM5_ESM.zip › Figure 3/3F/co-m0.png]

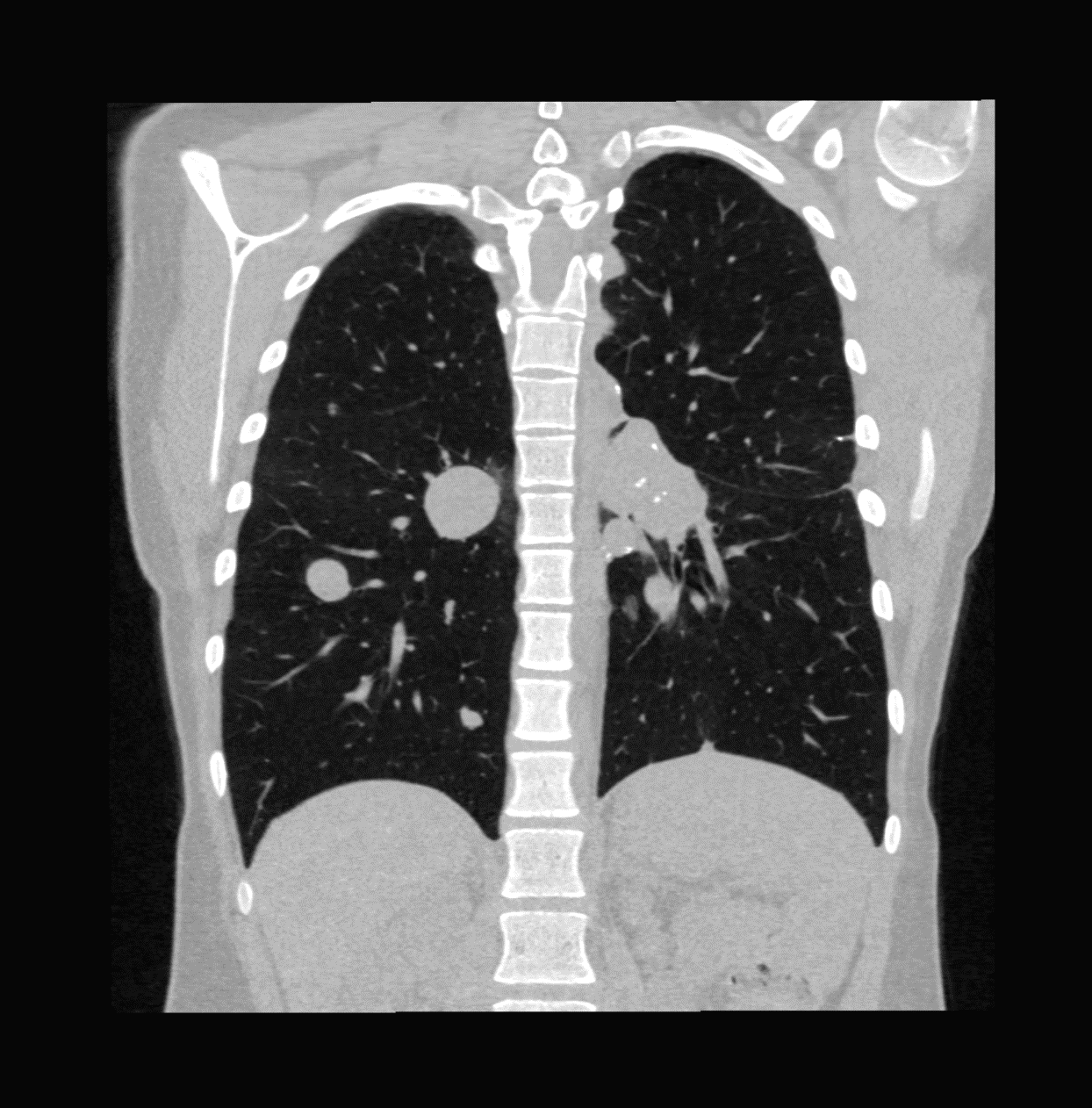

Supplement: Supplementary file 5 — Source data Fig. 3 [file 44321_2025_212_MOESM5_ESM.zip › Figure 3/3F/co-m5.png]

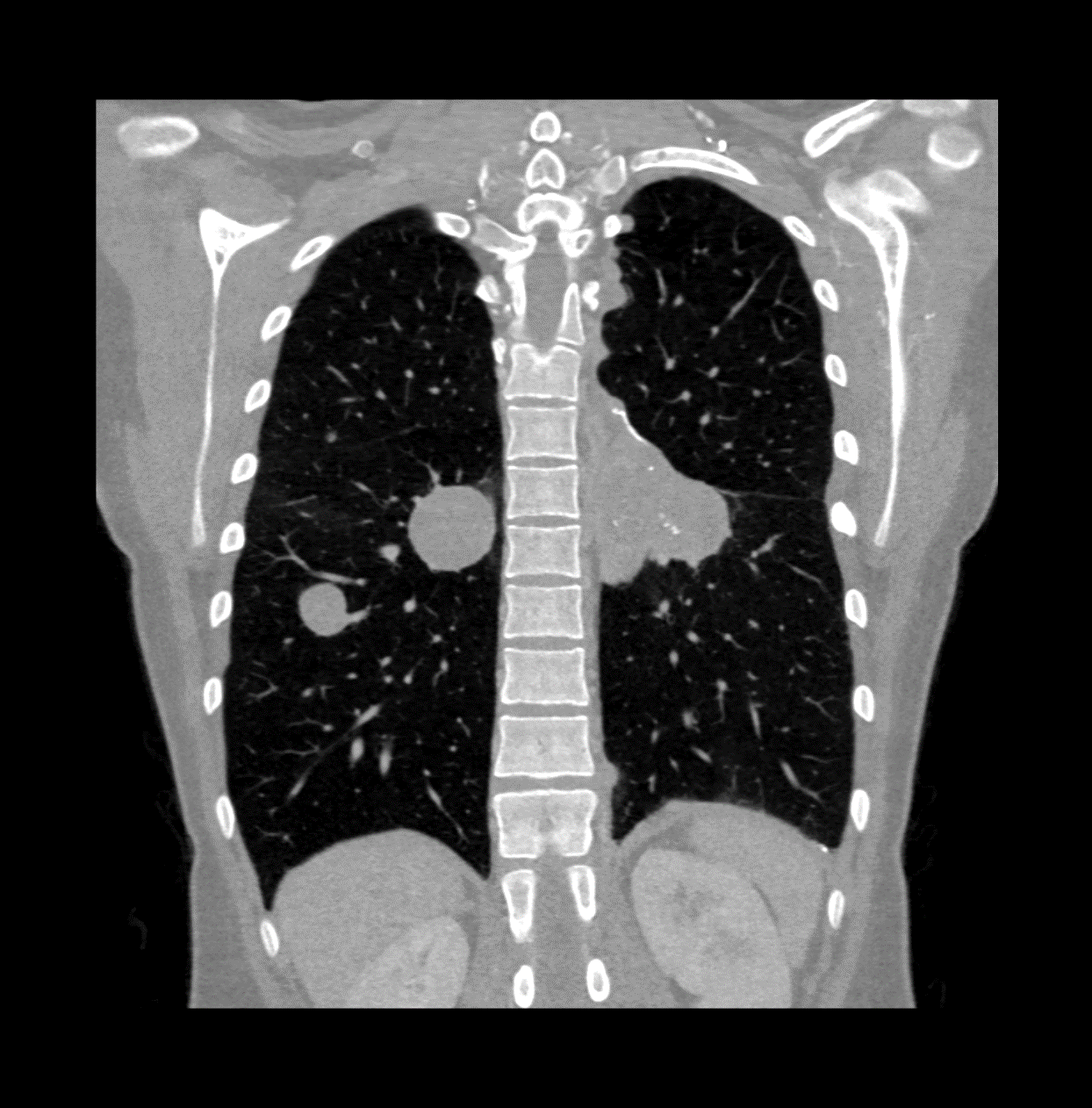

Supplement: Supplementary file 5 — Source data Fig. 3 [file 44321_2025_212_MOESM5_ESM.zip › Figure 3/3F/co-m7.png]

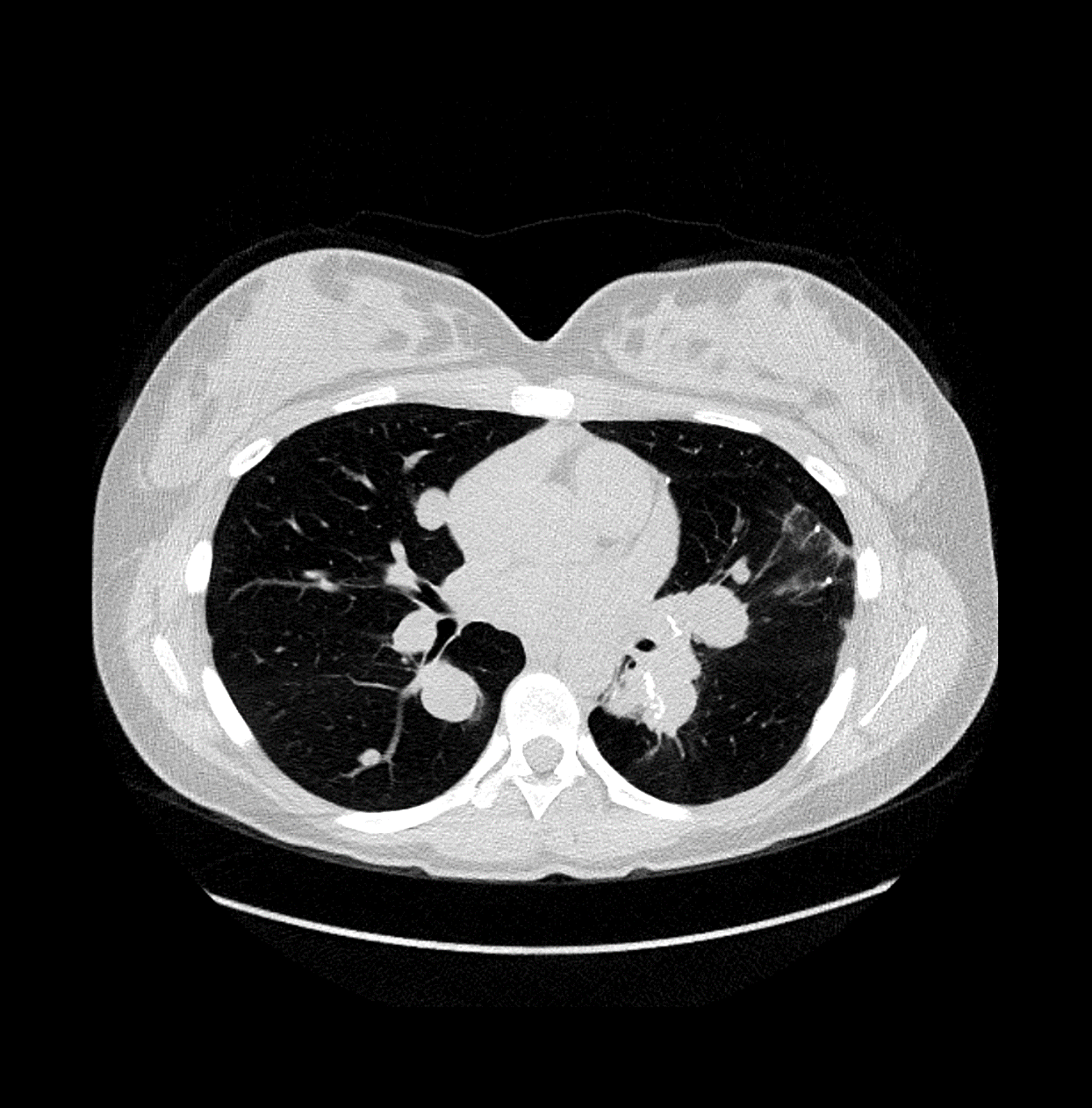

Supplement: Supplementary file 5 — Source data Fig. 3 [file 44321_2025_212_MOESM5_ESM.zip › Figure 3/3F/ax-m2.png]

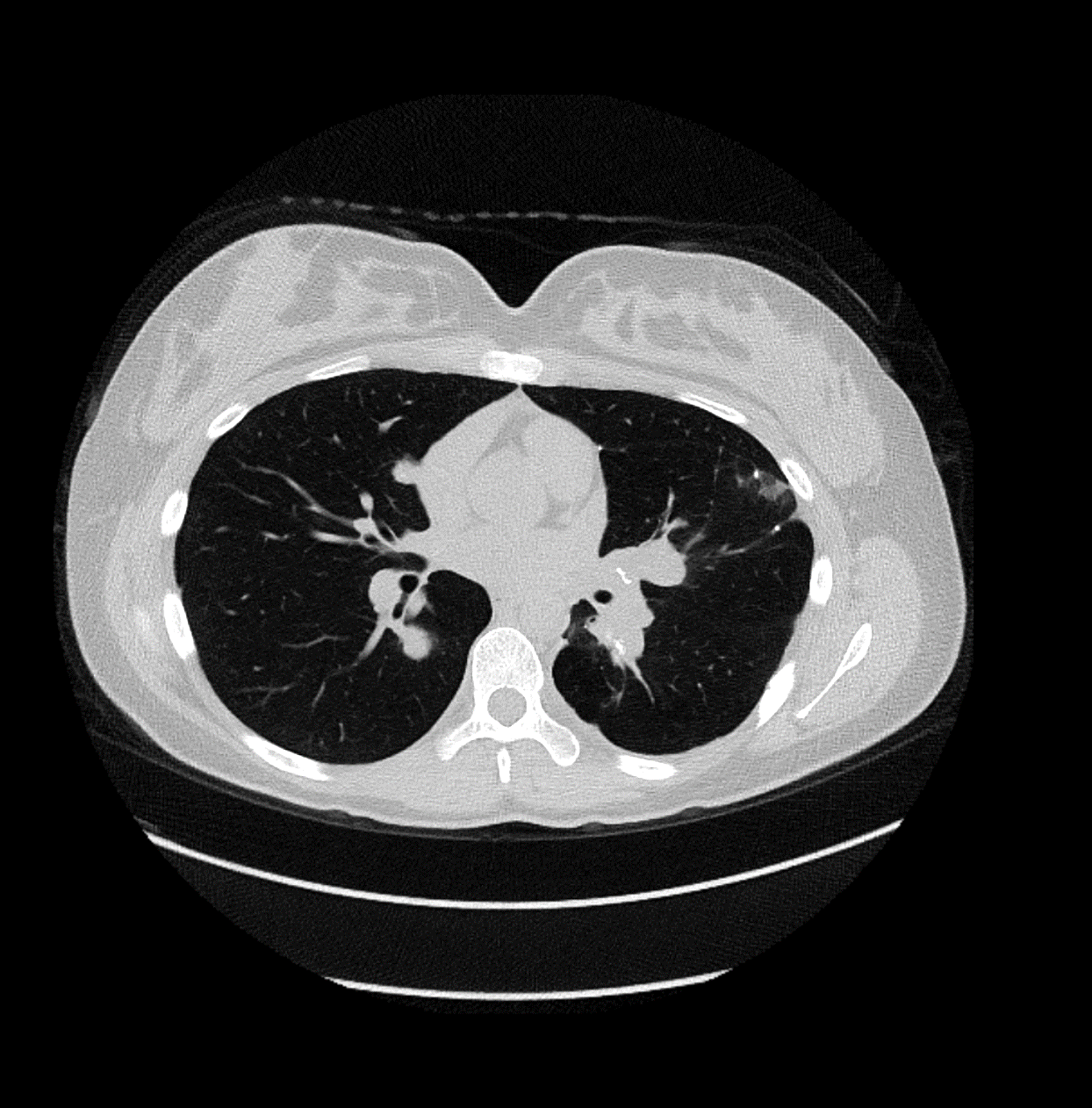

Supplement: Supplementary file 5 — Source data Fig. 3 [file 44321_2025_212_MOESM5_ESM.zip › Figure 3/3F/ax-m0.png]

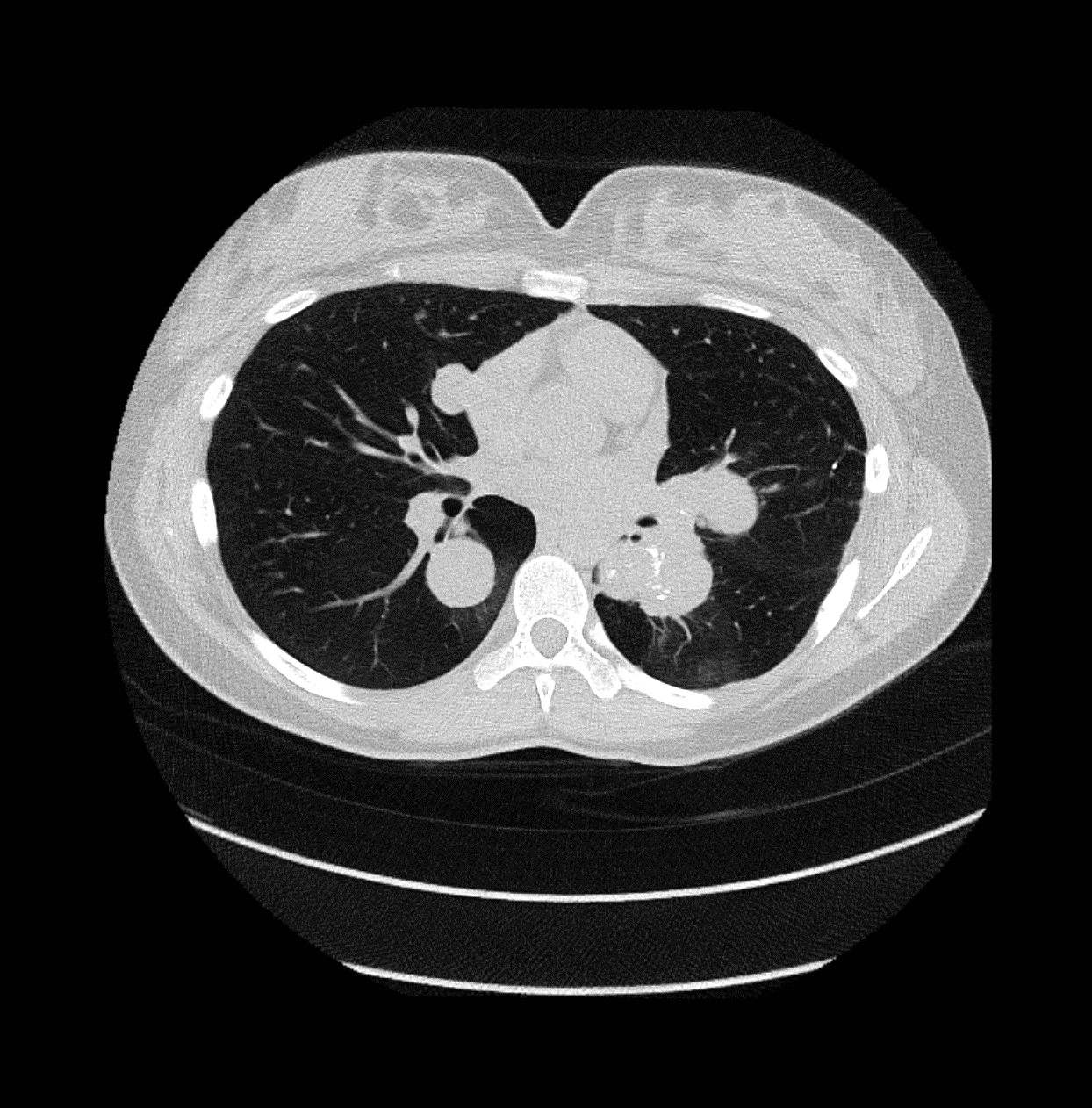

Supplement: Supplementary file 5 — Source data Fig. 3 [file 44321_2025_212_MOESM5_ESM.zip › Figure 3/3F/ax-m5.png]

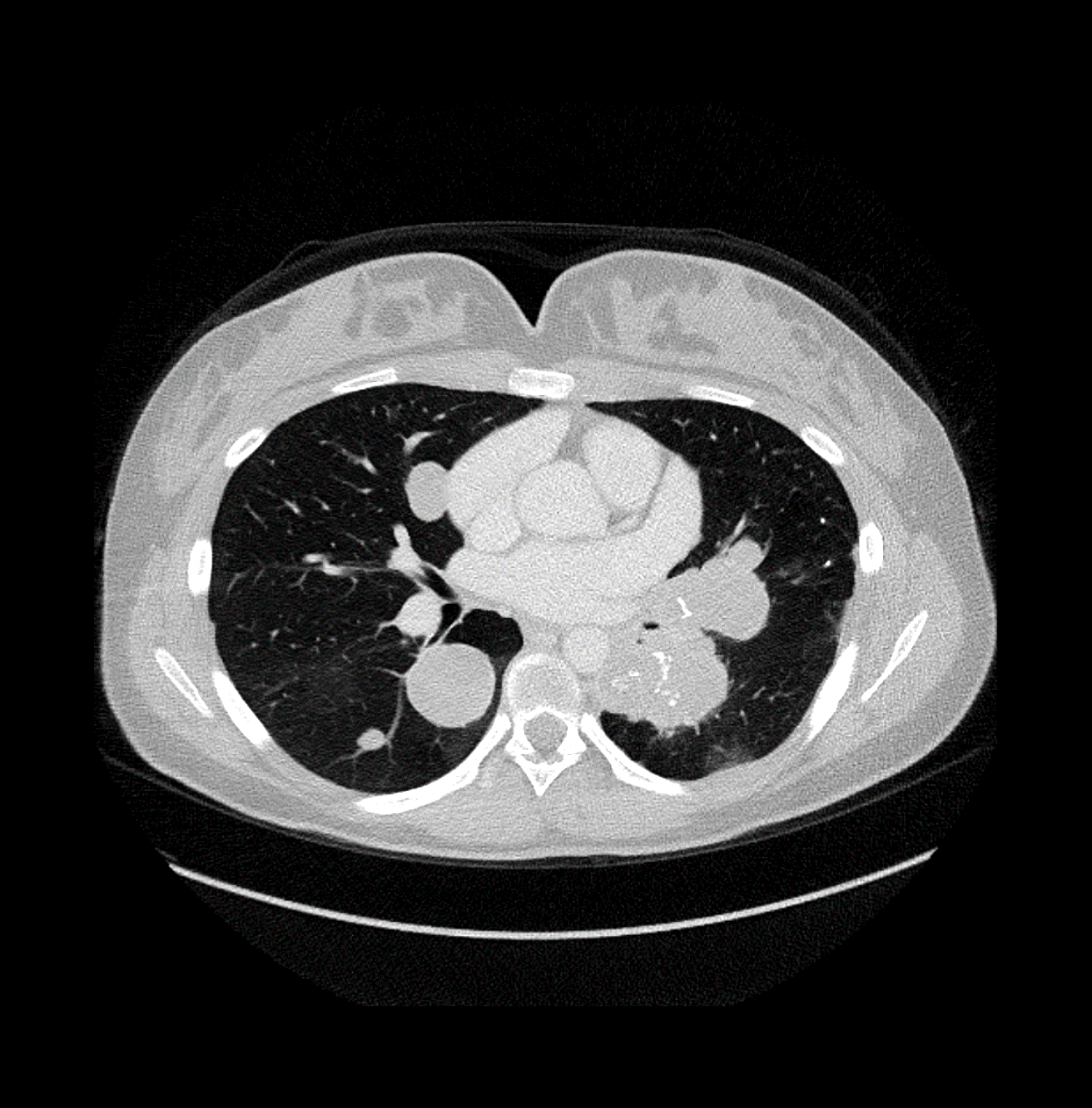

Supplement: Supplementary file 5 — Source data Fig. 3 [file 44321_2025_212_MOESM5_ESM.zip › Figure 3/3F/ax-m7.png]
