# Supplementary material for: Proteomics and personalized PDX models identify treatment for a progressive malignancy within an actionable timeframe
Source: EMBO Mol Med. 2025 Apr 1;17(4):625–44. doi: 10.1038/s44321-025-00212-8 (PMC11982353; doi:10.1038/s44321-025-00212-8)
Supplement: Supplementary file 7 — Expanded View Figures [file 44321_2025_212_MOESM7_ESM.pdf]

Expanded View Figures

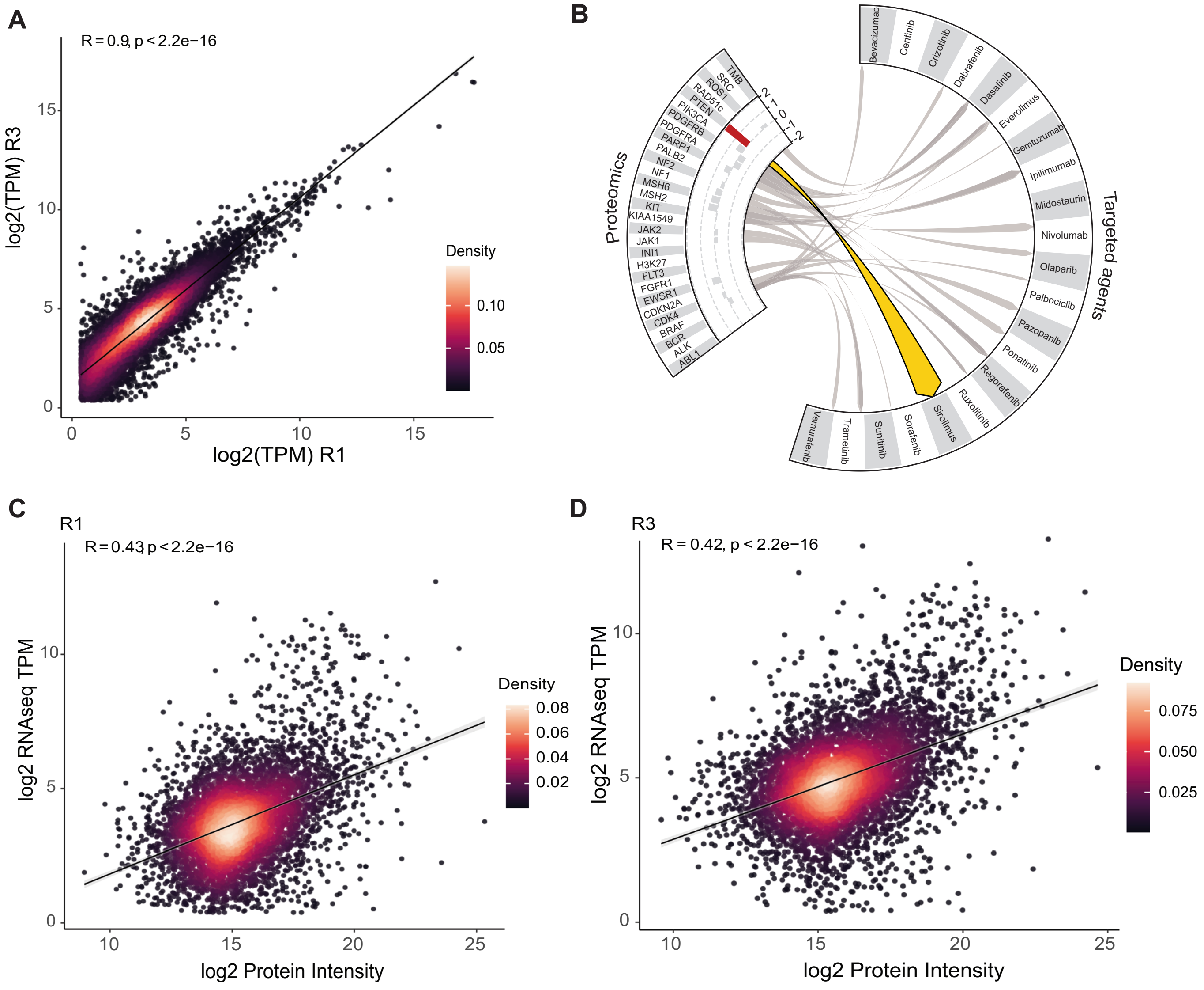

Figure EV1. Gene and protein expression in progressive SETTLE tumor.

(**A**) Correlation plot of gene expression from R1 and R3 with Pearson correlation coefficient r = 0.9, $p = 1e^{-1022}$. (**B**) Proteome changes do not suggest sensitivity to 21 established therapies. Circular plot showing the targeted proteomic analysis focusing on 29 proteins associated with 21 routinely used therapies in R3 lung nodules. Log2 fold-change values, calculated from the tumor vs normal comparison, are displayed on the proteomics side of the plot. Only associations for quantified proteins are shown. gray arrow: non-significant changes and associations; red arrow: proteome change counter indicative of drug sensitivity. (**C**, **D**) Scatter plot comparing protein intensities with gene expression in relapse R1 (**C**), with Pearson correlation coefficient r = 0.43, $p = 4.4e^{-261}$ and R3 (**D**), with Pearson correlation coefficient r = 0.42, $p = 2.4e^{-254}$.

© The Author(s)

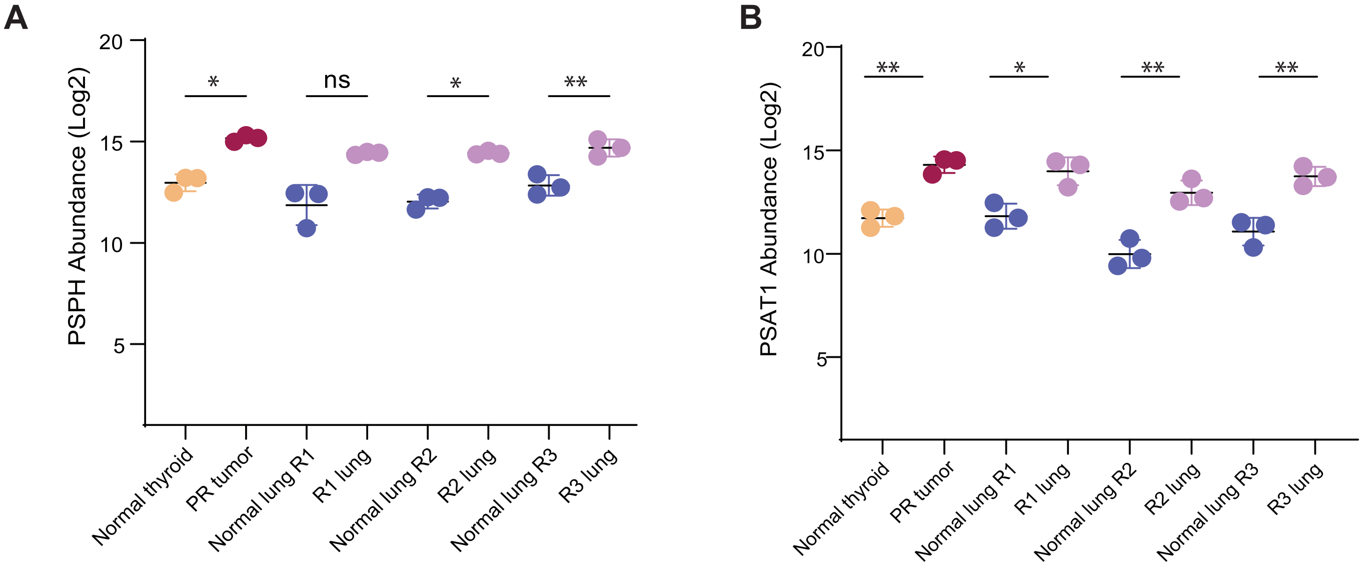

Figure EV2. Abundance of serine biosynthesis proteins in SETTLE.

(**A**, **B**) Increased abundance of serine biosynthesis proteins PSPH (**A**) and PSAT1 (**B**) in the primary tumor and lung metastases. Data shown represent three technical replicates for each group (mean ± s.d.), Bonferroni's multiple comparisons test, for PSPH: normal thyroid vs PR tumor *p(adjusted) = 0.0208, normal lung R1 vs R1 lung ^{ns}p(adjusted) = 0.1596, normal lung R2 vs R2 lung *p(adjusted) = 0.0201, normal lung R3 vs R3 lung **p(adjusted) = 0.0054; for PSAT: normal thyroid vs PR tumor **p(adjusted) = 0.0015, normal lung R1 vs R1 lung *p(adjusted) = 0.0142, normal lung R2 vs R2 lung **p(adjusted) = 0.0046, normal lung R3 vs R3 lung **p(adjusted) = 0.0048. Source data are available online for this figure.

 © The Author(s)

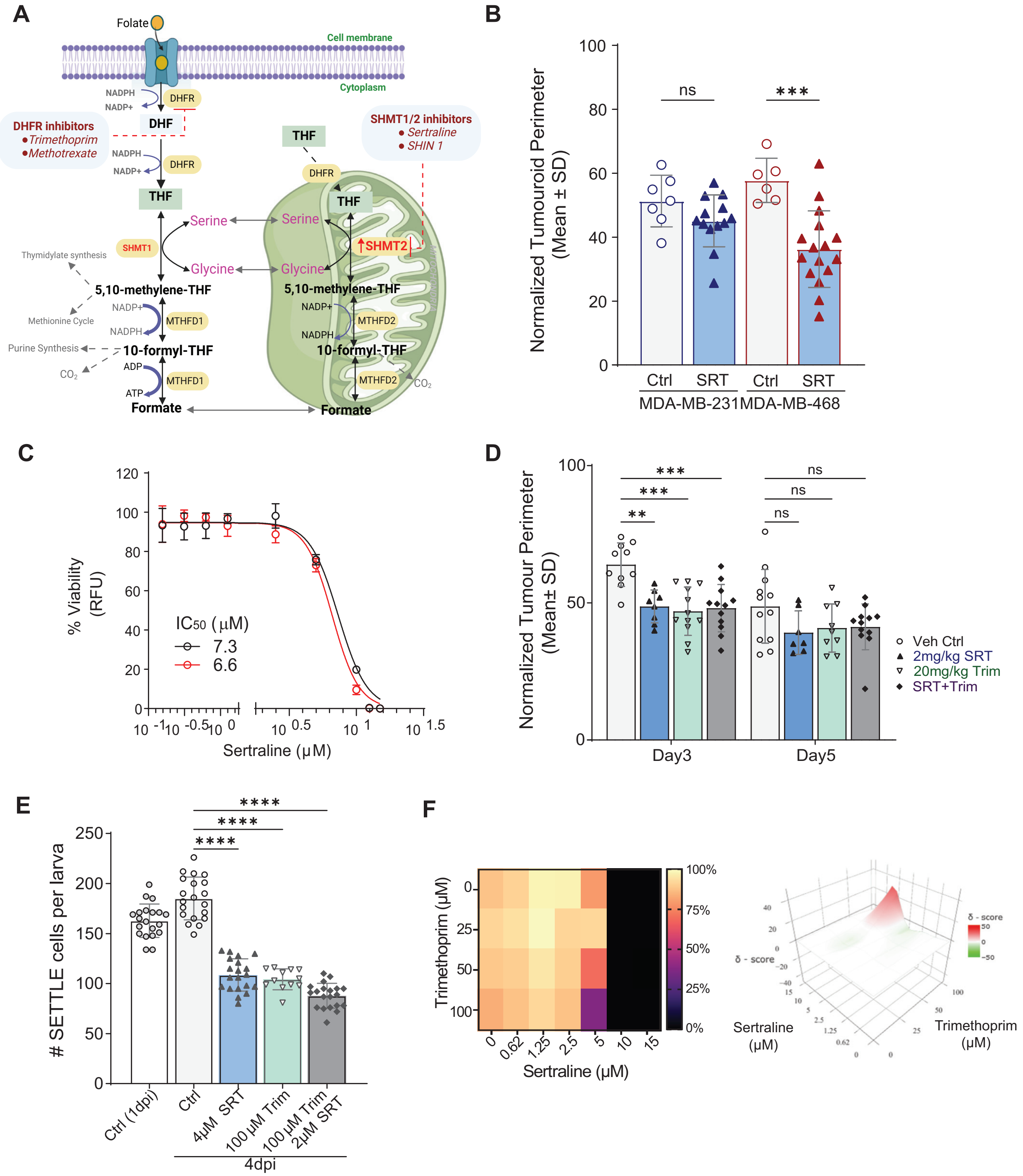
A
Folate
Cell membrane
Cytoplasm
NADPH
NADP+
DHFR
DHF
DHFR inhibitors
•Trimethoprim
•Methotrexate
THF
SHMT1/2 inhibitors
• Sertraline
• SHIN 1
Serine
Glycine
SHMT1
↑SHMT2
Thymidylate synthesis
5,10-methylene-THF
Methionine Cycle
MTHFD1
MTHFD2
Purine Synthesis
10-formyl-THF
ADP
ATP
CO_2
Formate
B
Normalized Tumouroid Perimeter (Mean ± SD)
ns
***
Ctrl
SRT
MDA-MB-231
MDA-MB-468
C
% Viability (RFU)
IC50 (µM)
7.3
6.6
Sertraline (µM)
D
Normalized Tumour Perimeter (Mean± SD)
**
***
ns
Veh Ctrl
2mg/kg SRT
20mg/kg Trim
SRT+Trim
Day3
Day5
E
SETTLE cells per larva
****
Ctrl (1dpi)
Ctrl
4µM SRT
100 µM Trim
100 µM Trim 2µM SRT
4dpi
F
Trimethoprim (µM)
Sertraline (µM)
100%
75%
50%
25%
0%
δ - score
Trimethoprim (µM)

Figure EV3. Sertraline and trimethoprim combination as a potential therapeutic approach for SETTLE.

(**A**) Schematic representation of the 1C metabolism pathway showing the significance of therapeutic inhibition of this pathway at DHFR and SHMT2 levels. Serine is converted to glycine by cytoplasmic SHMT1 (left) and mitochondrial SHMT2 (right). The 1C component sliced from serine is transferred to THF, generating methylene-THF. Therapeutic interventions, highlighted in red-dash lines (DHFR inhibitor: Trimethoprim, SHMT2 inhibitor: Sertraline), at two distant ends of this pathway stop the 1C unit being used for THF. THF is produced from folate and serves as a universal 1C acceptor. *DHF: dihydrofolate; THF: tetrahydrofolate; DHFR: dihydrofolate reductase; MFT: mitochondrial folate transporter; SHTMT1/2, serine hydroxymethyl transferase, cytosolic (1)/mitochondrial (2); MTHFD1: methylenetetrahydrofolate dehydrogenase 1; MTHFD2: methylenetetrahydrofolate dehydrogenase 2 (2-like)*. (**B**) Tumoroid perimeters at Day 4, normalized to Day 1, for CAM xenografts of MDA-MB-468 or MDA-MB-231 cells with and without treatment with sertraline (mean ± s.d., $n = 7$;13 tumors/group for MDA-MB-231, $n = 6$;16 tumors/group for MDA-MB-468, One-way ANOVA with Tukey's post hoc test ***$p = 0.0002$). (**C**) In vitro viability assays of NSG-PDX SETTLE cells subjected to treatment with sertraline. As plotted is the mean±s.d. for triplicate wells for each concentration of sertraline, for 2 independently conducted assays. (**D**) CAM engrafted with NSG-PDX SETTLE cells were untreated or treated with sertraline and/or trimethoprim at the indicated concentrations. The scatter bar graph depicts the tumoroid perimeters at days 3 and 5 normalized against day 1 (mean ± s.d., $n = 7$–12 tumors/group, two-way ANOVA with Dunnett's post hoc test, Veh Ctrl vs 2 mg/kg SRT **$p = 0.0041$, Veh Ctrl vs 20 mg/kg Trim ***$p = 0.0074$, Veh Ctrl vs SRT +Trim ***$p = 0.0074$). (**E**) NSG-PDX SETTLE cells engrafted in larval zebrafish were untreated or treated with sertraline (Sert) and/or trimethoprim (Trim). Scatter bar graphs depict the number of SETTLE cells per larva at 1 or 4 days post-implantation (dpi) (mean ± s.d., n(Ctrl) = 20; n(SRT) = 20; n(Trim) = 12; n(Trim+SRT) = 19 larval zebrafish/group, One-way ANOVA with Dunnet's post hoc test; ctrl vs 4 µM SRT ****$p = 0.0001$, ctrl vs 100 µM Trim ****$p = 0.0001$, ctrl vs 100 µM Trim+2µM SRT ****$p = 0.0001$). (**F**) In vitro viability assays of NSG-PDX SETTLE cells subjected to treatment with sertraline and/or trimethoprim shown as heat map viability plots (left) and the Highest Single Agent combinatorial drug synergy plots (d-score >10 indicative of synergistic effects) (right). Source data are available online for this figure.

© The Author(s)
